# Radiation‐induced extracellular vesicles from cancer‐associated fibroblasts drive oesophageal squamous cell carcinoma metastasis via the miR‐193a‐3p/PTEN/Akt pathway

**DOI:** 10.1002/ctm2.70483

**Published:** 2025-09-25

**Authors:** Yechun Pang, Tiantian Guo, Yue Zhou, Shanshan Jiang, Yida Li, Jianjiao Ni, Xiao Chu, Li Chu, Fangyu Chen, Xi Yang, Zhengfei Zhu

**Affiliations:** ^1^ Department of Radiation Oncology Fudan University Shanghai Cancer Center Shanghai China; ^2^ Department of Oncology, Shanghai Medical College Fudan University Shanghai China; ^3^ Shanghai Clinical Research Center for Radiation Oncology Shanghai China; ^4^ Shanghai Key Laboratory of Radiation Oncology Shanghai China; ^5^ Department of Radiation Oncology Fudan University Zhongshan Hospital Shanghai China; ^6^ Institute of Thoracic Oncology Fudan University Shanghai China

**Keywords:** ESCC, extracellular vesicles, metastasis, radiation

## Abstract

**Background:**

The recurrence and metastasis of oesophageal squamous cell cancer (ESCC) following radiation therapy are major treatment challenges. Cancer‐associated fibroblasts (CAFs) are key in the ESCC microenvironment, yet their role in post‐radiation recurrence remains unclear.

**Materials and Methods:**

KYSE150 ESCC cells were co‐implanted with non‐irradiated (0 Gy) or irradiated (8 Gy) CAFs in nude mice. CAF‐derived extracellular vesicles (EVs) were isolated via differential centrifugation and analysed by electron microscopy and immunoblotting. Transwell assays evaluated EVs' effects on ESCC cell migration and invasion in vitro. RNA sequencing identified differentially expressed microRNAs, and functional experiments verified the role of miR‐193a‐3p. Plasma samples from 32 ESCC patients and tissue samples from 76 ESCC patients were analysed for miR‐193a‐3p expression.

**Results:**

Irradiated CAFs promoted the lung metastasis of ESCC cells in vivo, and their EVs enhanced ESCC cell invasion, migration and metastasis. Elevated miR‐193a‐3p levels in EVs from irradiated CAFs increased miR‐193a‐3p expression in ESCC cells. This effect was effectively attenuated by RNase and Triton X‐100 (degrading microRNAs encapsulated in EVs), or GW4869 (inhibiting EVs biogenesis and secretion)—indicating that miR‐193a‐3p functions in an EV‐dependent manner. Knockdown of miR‐193a‐3p diminished the invasion, migration and epithelial–mesenchymal transition (EMT)‐promoting activities of CAF‐derived EVs. Luciferase assays confirmed PTEN as a target of miR‐193a‐3p; miR‐193a‐3p overexpression decreased PTEN and increased p‐Akt expression. In vivo, coinjection of miR‐193a‐3p‐knockdown CAFs with KYSE150 ESCC cells resulted in smaller tumours, fewer lung metastases, increased PTEN and E‐cadherin, and decreased p‐Akt and Snail expression. Clinically, radiation increased plasma exosomal miR‐193a‐3p levels, and high miR‐193a‐3p expression was correlated with shorter survival, identifying miR‐193a‐3p as an independent predictor of poor prognosis in ESCC patients.

**Conclusion:**

EVs from irradiated CAFs promote ESCC metastasis via the miR‐193a‐3p‐mediated PTEN/Akt signalling pathway. Targeting this EVs‐mediated interaction represents a promising strategy for improving ESCC radiotherapy outcomes.

**Key points:**

- The poor prognosis of oesophageal squamous cell carcinoma (ESCC) is largely driven by recurrence and metastasis following radiation therapy.
- Irradiated cancer‐associated fibroblasts (CAFs) drive ESCC recurrence and metastasis through extracellular vesicles (EVs), highlighting their critical role in the post‐radiation tumor microenvironment.
- CAF‐derived EVs deliver miR‐193a‐3p to ESCC cells, suppressing PTEN and activating Akt signaling, thereby enhancing invasion, migration, epithelialmesenchymal transition (EMT), and metastatic potential.
- High plasma exosomal miR‐193a‐3p levels predict poor prognosis in ESCC patients and may guide therapeutic strategies after radiotherapy.

## Graphical abstract

1. miR‐193a‐3p is upregulated in extracellular vesicles (EVs) derived from irradiated cancer‐associated fibroblasts (CAFs).
2. Irradiated CAF‐derived EV miR‐193a‐3p promotes the metastasis and epithelial–mesenchymal transition (EMT) of oesophageal squamous cell cancer (ESCC) cells.
3. miR‐193a‐3p promotes ESCC metastasis through PTEN/Akt‐mediated EMT.

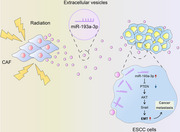

## 1 INTRODUCTION

Oesophageal cancer is a highly aggressive malignancy and the sixth leading cause of cancer‐related mortality worldwide.^1^ Oesophageal squamous cell carcinoma (ESCC) accounts for ∼90% of cases.^2^ ESCC is often diagnosed in locally advanced stages, where radiotherapy plays a key role in the management of ESCC patients. Although most patients initially respond, disease progression inevitably occurs, with over 50% developing distant recurrence within 2 years.^3^, ^4^ Radiotherapy directly kills tumour cells and stimulates immune responses by releasing tumour antigens and also alters the tumour microenvironment (TME), potentially promoting immune evasion.^5^, ^6^ For example, radiotherapy induces immunosuppressive cytokines (e.g., TGF‐β, IL‐10) and chemokines that recruit myeloid inflammatory cells, which contribute to recurrence by suppressing antitumour immunity.^7^ Furthermore, radiotherapy also affects non‐immune TME components,^8^, ^9^, ^10^ which significantly influence tumour relapse.^11^, ^12^, ^13^ Radiation‐induced alterations of non‐immune cells impact both local recurrence and metastasis, underscoring the need to understand their role in improving treatment efficacy.

Cancer‐associated fibroblasts (CAFs) are key non‐immune components of the TME and play a crucial role in cancer progression by supporting tumour growth.^14^, ^15^ Increasing evidence suggests that CAFs facilitate tumour progression through direct interactions with tumour cells.^16^, ^17^, ^18^ In ESCC, CAFs have been implicated in regulating the malignant behaviours of tumour cells and their response to therapy.^19^, ^20^, ^21^, ^22^ Given that CAFs reside within the TME, they are inevitably exposed to radiation during tumour radiotherapy. Radiation‐induced stress may alter CAF function and disrupt CAF‐cancer cell interactions.^23^ However, the role of CAFs in ESCC recurrence post‐radiotherapy remains unexplored.

Extracellular vesicles (EVs) mediate intercellular communication within the TME by transferring signalling molecules.^24^, ^25^, ^26^ EVs include exosomes (50–120 nm), which originate from the endocytic pathway, and microvesicles (50–1500 nm) which bud from plasma membranes.^27^ These vesicles carry proteins, lipids, metabolites, mitochondrial DNA, messenger RNA and non‐coding RNAs.^28^, ^29^ MicroRNAs (miRNAs) are key EV cargo with crucial roles in tumour regulation. CAFs are a major source of EVs within the TME.^30^ Exosome secretion is a key mechanism by which CAFs influence cancer cells,^31^, ^32^ such as modulating metabolic reprogramming via reduced oxygen consumption.^33^ In colorectal cancer, CAF‐derived exosomes enhance cancer cell radioresistance by preventing DNA damage and inhibiting apoptosis.^34^ However, the role of EVs from irradiated CAFs in ESCC has not yet been studied.

This study aims to investigate the role of irradiated CAFs in ESCC recurrence following radiotherapy. Our findings demonstrated that irradiated CAFs promote ESCC metastasis via EV‐mediated transfer of miRNAs from CAFs to cancer cells and identify miR‐193a‐3p/PTEN/Akt as the key pathway underlying this effect.

## 2 MATERIALS AND METHODS

### 2.1 Tissue and plasma specimens

Fresh ESCC tissues for in situ hybridisation were obtained from 76 patients who underwent surgical resection at Fudan University Shanghai Cancer Centre between January 2014 and June 2014. The median follow‐duration was 35.6 months (interquartile range 23.5–49.6 months). Two senior pathologists confirmed ESCC diagnoses. Tumours were staged according to the eighth edition of the American Joint Committee on Cancer TNM classification. Participants were eligible for inclusion if they had not received preoperative chemotherapy and/or radiation therapy. The clinicopathological characteristics of the included patients are summarised in Supporting Information Table 1.

Blood samples were collected from 32 patients with cytologically or histopathologically confirmed ESCC who underwent concurrent definitive chemoradiation therapy at Fudan University Shanghai Cancer Center between February 2023 and June 2023. Samples were collected 1 day before treatment and 14 days after the initiation of concurrent chemoradiation therapy. Written informed consent was obtained from all patients. This study was conducted in accordance with the Declaration of Helsinki and was approved by the Ethics Committee of Fudan University Shanghai Cancer Center (no. 0504323‐4‐2307E).

### 2.2 Cell culture

Human ESCC cell lines KYSE150 and TE‐1 were obtained from the Shanghai Cell Bank of Chinese Academy of Sciences and maintained in RPMI 1640 medium supplemented with 10% foetal bovine serum (FBS; Gibco, Thermo Fisher Scientific, Inc.) in a humidified incubator (37°C, 5% CO_2_). All cultures were tested for *Mycoplasma* contamination before use.

### 2.3 Isolation and culture of CAFs

CAFs were isolated from the central region of ESCC tumour tissue. After surgical resection, the samples were washed in phosphate‐buffered saline (PBS) containing 1% antibiotic–antimycotic (Life Technologies, Thermo Fisher Scientific). The tissues were then minced and digested for 2 h at 37°C with shaking using collagenase (Sigma‐Aldrich) and diluted in Dulbecco's modified Eagle's medium (DMEM). After centrifugation, cell pellets were resuspended in DMEM supplemented with 10% FBS and filtered through a 100‐µm cell strainer. The cells were then cultured in DMEM/F12 medium supplemented with 10% FBS at 37°C with 5% CO_2_. Fibroblast purity was assessed by immunofluorescent staining, and CAFs were used for experiments up to 10 passages.

### 2.4 Preparation of conditioned medium from irradiated CAFs

CAFs were inoculated into 25 cm^2^ cell culture flasks and exposed to X‐rays using a Small Animal Radiation Research Platform (Primus‐Hi, Siemens Healthineers). When cell confluency reached 70%–80%, cells were irradiated with a single dose of 8 Gy, while control cells received sham irradiation (0 Gy). After irradiation, the culture medium was replaced with DMEM/F12 medium containing 10% vesicle‐depleted FBS (VivaCell). After 72 h, the conditioned medium (CM) from sham or irradiated CAFs was collected, sterilised and normalised to an equal number of cells.

### 2.5 EV isolation and characterisation

EVs were isolated from CAF culture‐conditioned supernatant and plasma via differential ultracentrifugation.^35^ Exosomes were deposited on the copper grid, which was touched onto a drop of 2% uranyl acetate for 1 min before air‐drying and imaging by transmission electron microscopy (JEM‐1200EX, JEOL). NanoSight NS300 nanoparticles (Malvern Instruments) were used to determine EV size distribution. The expression of EV markers (CD63, TSG101 and CD9) was confirmed via Western blot. EVs were stored at −80°C. For in vitro experiments, 15 µg/mL EVs were premixed with a culture medium.

### 2.6 EV miRNA sequencing

EV miRNA sequencing was performed by Lc‐Bio Technologies Co., Ltd. Briefly, total RNA was extracted from EVs, and a small RNA library was constructed using the TruSeq Small RNA Library Preparation Kit (Illumina). The libraries were sequenced on the HiSeq 2500 platform with 50‐bp single‐end reads. Differentially expressed genes (fold change ≥1.5 with *p*  < .05) were analysed using DESeq2.

### 2.7 Cell transfections

hsa‐miR‐193a‐3p mimics, inhibitors and negative controls were purchased from RiboBio Co., Ltd. The mature hsa‐miR‐193a‐3p sequence was (5′‐AACUGGCCUACAAAGUCCCAGU‐3′). PTEN small interfering RNA (siRNA) and scramble siRNA were purchased from GenePharma Co., Ltd. Transfection of siRNA, miRNA mimics and miRNA inhibitors was performed using Lipofectamine RNAiMAX (Thermo Fisher Scientific). Tumour cells were transfected with PTEN overexpression plasmid (pcDNA3.1‐PTEN) or the negative control vectors with Lipofectamine 3000 (Thermo Fisher Scientific). CAFs were infected with miR‐193a‐3p‐knockdown lentivirus or control lentivectors (GeneChem Co., Ltd.). The primer sequences are listed in Supporting Information Table 2.

### 2.8 Western blotting

Proteins were extracted using RIPA lysis buffer containing protease and phosphatase inhibitor cocktail (Thermo Fisher Scientific). Protein concentrations were determined using a BCA Protein Assay Kit (Beyotime). Each sample equivalent of 20 µg total protein was separated via sodium dodecyl‐sulphate polyacrylamide gel electrophoresis and transferred onto polyvinylidene fluoride membranes. After blocking with 5% bovine serum albumin in tris‐buffered saline with Tween20, polyvinylidene fluoride membranes were immunoblotted overnight at 4°C with primary antibodies. Horseradish peroxidase‐conjugated secondary antibodies were used for detection, and immunoreactive bands were visualised using Pierce ECL Western Blotting Substrate (Thermo Fisher Scientific). The primary antibodies are summarised in Supporting Information Table 3.

### 2.9 RNA extraction and quantitative reverse transcription polymerase chain reaction

Total RNA was extracted from cells using TRIzol reagent (Invitrogen, Thermo Fisher Scientific). EV‐derived RNA extraction was performed using the Total Exosome RNA and Protein Isolation Kit (Invitrogen). Complementary DNA synthesis for mRNAs was performed using the PrimeScript RT Reagent Kit (Takara Bio), While miRNA reverse transcription was conducted using the Mir‐X miRNA First‐Strand Synthesis Kit (Takara). Quantitative reverse transcription polymerase chain reaction (qRT‐PCR) was performed in triplicate on an Applied Biosystems 7500 Fast Real‐Time PCR System (Thermo Fisher Scientific) with the TB Green kit (Takara Bio). Relative mRNA expression levels were analysed using the 2^−ΔΔCT^ method, with GAPDH as an internal control. For miRNA, U6 snRNA was used as an internal control for cellular samples, while cel‐miR‐39 was used as an external control for EV‐derived miRNA detection. The primers for target genes are listed in Supporting Information Table 2.

### 2.10 RNA stability assays

Actinomycin D (AbMole) was added to TE‐1 cells transfected with miR‐193a‐3p mimics or NC mimic in a 6‐well plate at a concentration of 5 ug/mL for 0, 3 and 6 h intervals. After extraction, total RNA was reverse‐transcribed and evaluated using RT‐qPCR. As a means of measuring RNA abundance, the average Ct value at each time point was set to 0 h.

### 2.11 Animal models

Female nude mice (BALB/c‐nu, 5 weeks old) were purchased from Shanghai Slack Laboratory Animal Co. Ltd. and maintained in a specific pathogen‐free facility under a 12‐h light–dark schedule. Sterile food and water were provided ad libitum. All in vivo experiments were approved by the Institutional Animal Care and Use Committee of Fudan University Shanghai Cancer Center. Animals were assigned to experimental groups using simple randomisation.

For co‐implantation models, mixed cells (1 × 10^6^ KYSE150 cells and 5 × 10^5^ CAFs) were subcutaneously inoculated into the right flank of 5‐week‐old nude mice. Tumour size was measured every 5 days, and tumour volume was calculated using the formula: volume = length × width^2^/2. Mice were euthanised on day 40 post‐implantation. The number of metastatic lung nodules was determined using haematoxylin–eosin (H&E) staining and quantified under a microscope.

To evaluate the effects of EVs on ESCC metastasis, a mouse lung metastasis model was established via tail–vein injection of 1 × 10^6^ KYSE150 cells (in  .1 mL PBS; *n* =  8 per group) with the indicated EVs (10 µg). Additional EV injections (10 µg) were injected into tail veins every 7 days. After six injections, mice were euthanised on the third day after the final injection, and the number of metastatic nodules was quantified.

### 2.12 Statistical analysis

All statistical analyses were performed using GraphPad Prism 8.0 (GraphPad Software). Data are expressed as the mean  ±  standard deviation (SD) from three independent experiments. Comparisons between two groups were assessed using a two‐sided unpaired Student's *t*‐test, while paired data were analysed using a paired *t*‐test. Differences among three or more groups were assessed using one‐way analysis of variance followed by Bonferroni's multiple comparison test. Tumour growth data were calculated using two‐way analysis of variance for repeated measures. Overall survival (OS), disease‐free survival (DFS) and distant metastasis‐free survival (DMFS) were estimated using the Kaplan–Meier method and compared with the log‐rank test. Univariable and multivariable Cox proportional hazard models were used to determine hazard ratios and 95% confidence intervals. A two‐sided *p* value of <.05 was considered statistically significant.

## 3 RESULTS

### 3.1 EVs derived from irradiated CAFs promote ESCC metastasis

**FIGURE 1:**
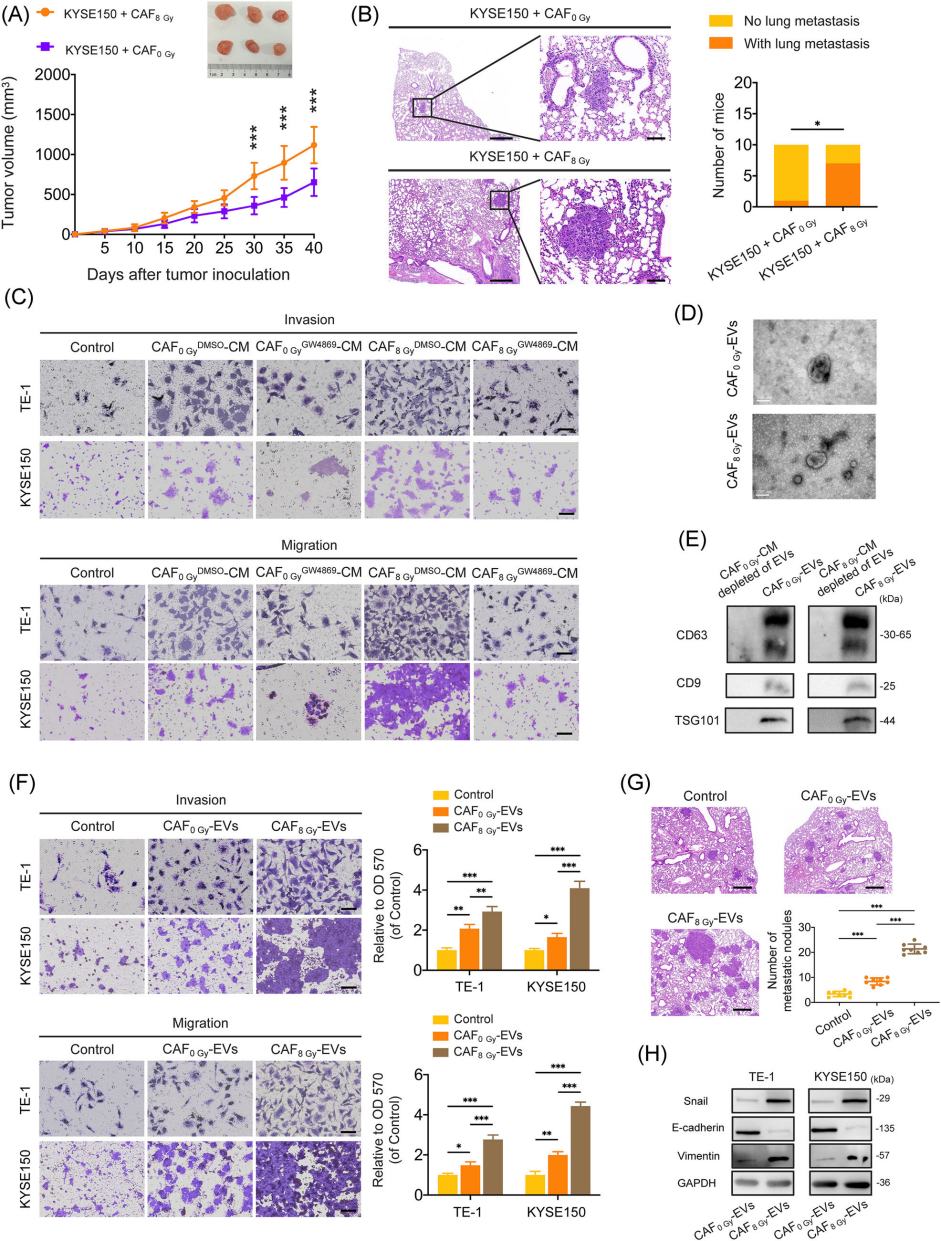
Extracellular vesicles (EVs) derived from irradiated cancer‐associated fibroblasts (CAFs) promoted the metastasis of oesophageal squamous cell cancer (ESCC). (A) Mixed cells (KYSE150 + CAF_0 Gy_ or KYSE150 + CAF_8 Gy_) were subcutaneously inoculated into the right flank area of 5‐week‐old nude mice (*n* = 10 mice per group). Tumour volumes were recorded and calculated. The tumours were excised on the 40th day after inoculation. Representative tumour images in the KYSE150 + CAF_0 Gy_ and KYSE150 + CAF_8 Gy_ groups are shown. (B) Lung metastasis formation was observed on the 40th day after inoculation. Representative haematoxylin–eosin (H&E) staining images of metastatic lung nodules in the KYSE150 + CAF_0 Gy_ and KYSE150 + CAF_8 Gy_ groups are shown (left), and lung metastasis in KYSE150 + CAF_0 Gy_ and KYSE150 + CAF_8 Gy_ groups was analysed with the Fisher's exact test (right). Scale bar: 500 µm (left) and 100 µm (right). (C) TE‐1 and KYSE150 cells were treated with controls, conditioned medium (CM) from DMSO‐treated CAF_0 Gy_, CM from GW4869‐treated CAF_0 Gy_, CM from DMSO‐treated CAF_8 Gy_ or CM from GW4869‐treated CAF_8 Gy_ for 24 h. TE‐1 and KYSE150 cells were then harvested for the Transwell assay (crystal violet staining). The absorbance of eluted crystal violet was read at 570 nm. Scale bar: 50 µm. (D) Transmission electron microscopy images of CAF_0 Gy_‐EVs and CAF_8 Gy_‐EVs. Scale bar: 100 nm. (E) Western blot analysis for CD63, CD9 and TSG101 of CAF_0 Gy_‐EVs, CAF_8 Gy_‐EVs, and supernatant with EVs depleted by ultracentrifugation (CAF_0 Gy_‐CM depleted of EVs, CAF_8 Gy_‐CM depleted of EVs). (F) Effects of CAF_0 Gy_‐EVs and CAF_8 Gy_‐EVs on the invasion and migration abilities of TE‐1 and KYSE150 cells according to Transwell assay (crystal violet staining). The absorbance of eluted crystal violet was read at 570 nm. Scale bar: 50 µm. (G) Effects of CAF_0 Gy_‐EVs and CAF_8 Gy_‐EVs on the formation of metastatic lung nodules (*n* = 8 mice per group). Representative H&E staining photographs of metastatic lung nodules are shown, and the numbers of metastatic lung nodules were calculated. Scale bar: 500 µm. (H) Western blot analysis for epithelial–mesenchymal transition (EMT) markers (Snail, Vimentin and E‐cadherin) in TE‐1 and KYSE150 cells treated with CAF_0 Gy_‐EVs and CAF_8 Gy_‐EVs. Data are presented as the mean ±  standard deviation (SD). *p < .05, **p < .01, ***p < .001.

To evaluate the impact of irradiated CAFs on ESCC progression, we isolated CAFs from tumour cells. CAFs isolated from ESCC tissues were characterised by alpha‐smooth muscle actin, fibroblast‐specific protein and fibroblast activation protein via immunofluorescence (Supporting Information Figure 1A). ESCC cells (KYSE150) were then co‐implanted subcutaneously in nude mice with either sham‐irradiated CAFs (CAF_0 Gy_) or irradiated CAFs (CAF_8 Gy_; Figure 1A). After 40 days, spontaneous lung metastases were significantly more frequent in the KYSE150 + CAF_8 Gy_ group than in the KYSE150 + CAF_0 Gy_ group (7/10 vs. 1/10; *p* = .020; Figure 1B). To further confirm the pro‐metastatic role of irradiated CAF, TE‐1 and KYSE150 cells were treated with CM from CAF_0 Gy_ (CAF_0 Gy_‐CM) and CAF_8 Gy_ (CAF_8 Gy_‐CM). CAF_0 Gy_‐CM treatment significantly enhanced ESCC cell invasion and migration. Notably, CAF_8 Gy_‐CM induced more pronounced effects (Figure 1C and Supporting Information Figure 1B), supporting that irradiated CAFs promote ESCC metastasis.

EVs have been reported to mediate intercellular communication between CAFs and cancer cells.^24^ The EV secretion inhibitor GW4869 abolished the pro‐invasive and pro‐migratory effects of CAF_8 Gy_‐CM on ESCC cells (Figure 1C and Supporting Information Figure 1B), implicating EVs in irradiated CAF‐mediated ESCC metastasis. EVs were then purified from CAF_0 Gy_‐CM and CAF_8 Gy_‐CM and the structural nature of isolated EVs was confirmed by transmission electron microscopy and nanosight tracking analysis (Figure 1D and Supporting Information Figure 1C). Western blot analysis confirmed the presence of exosomal markers (Figure 1E). Fluorescence labelling with PKH67 demonstrated EV uptake by ESCC cells (Supporting Information Figure 1D). Notably, functional analysis revealed that CAF_8 Gy_‐EVs significantly enhanced ESCC cell migration and invasion compared to CAF_0 Gy_‐EVs (Figure 1F). In vivo imaging of mice tissues injected with DiR‐labelled CAF_0 Gy_‐EVs or CAF_8 Gy_‐EVs further confirmed the absorption of EVs by ESCC tumour tissues (Supporting Information Figure 1E). In a lung metastasis model of ESCC using KYSE150 cells, intravenous injection of CAF_0 Gy_‐EV promoted lung metastasis, whereas CAF_8 Gy_‐EV treatment significantly increased the number of metastatic nodules (Figure 1G), suggesting the pro‐metastatic role of irradiated CAF‐derived EVs.

Since epithelial–mesenchymal transition (EMT) is critical for cancer metastasis, we examined EMT marker expression in EV‐treated ESCC cells in vitro or in ESCC metastatic lesions in vivo. CAF_8 Gy_‐EV treatment significantly increased mesenchymal markers (Vimentin and Snail) and decreased E‐cadherin expression in KYSE150 and TE‐1 cells (Figure 1H), as well as in metastatic ESCC tumours (Supporting Information Figure 1F), as compared to CAF_0 Gy_‐EVs treatment, suggesting that irradiated CAF‐derived EVs promote the EMT of ESCC cells

### 3.2 miR‐193a‐3p is upregulated in EVs derived from irradiated CAFs and could be transferred as EV cargo

**FIGURE 2:**
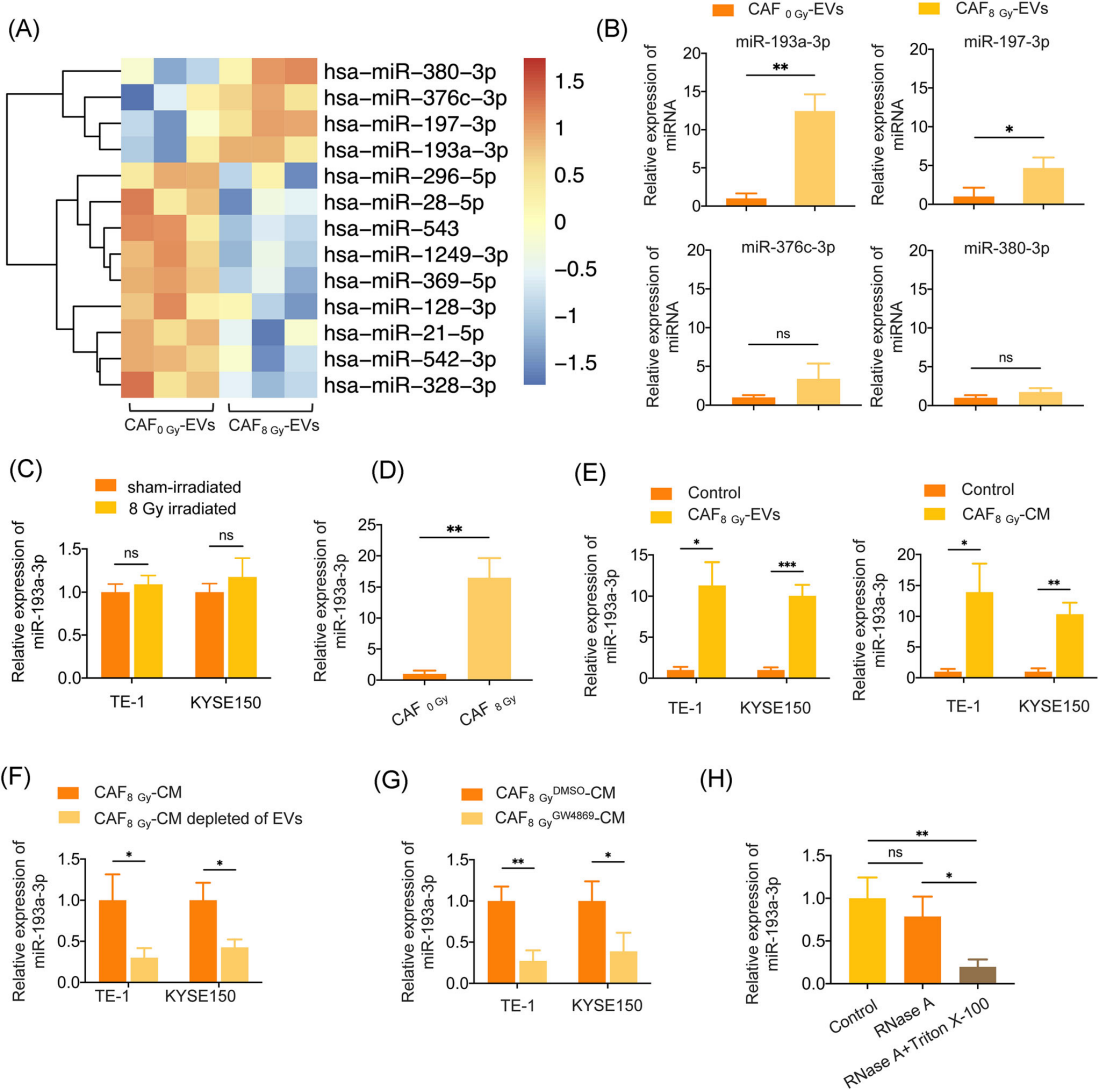
miR‐193a‐3p was upregulated in extracellular vesicles (EVs) derived from irradiated cancer‐associated fibroblasts (CAFs) and could be transferred to oesophageal squamous cell cancer (ESCC) cells via EVs. (A) Heatmap of differentially expressed (*p* < .05) miRNAs between CAF_0 Gy_‐EVs and CAF_8 Gy_‐EVs according to miRNA sequencing. (B) Relative expression of miR‐193a‐3p, miR‐197a‐3p, miR‐376c‐3p and miR‐380‐3p in CAF_0 Gy_‐EVs and CAF_8 Gy_‐EVs according to quantitative reverse transcription polymerase chain reaction (qRT‐PCR). (C) Relative expression of miR‐193a‐3p in sham‐irradiated and 8 Gy irradiated ESCC cells according to qRT‐PCR. (D) Relative expression of miR‐193a‐3p in CAF_0 Gy_ and CAF_8 Gy_ according to qRT‐PCR. (E) Relative expression of miR‐193a‐3p in TE‐1 and KYSE150 cells treated with phosphate‐buffered saline (PBS), CAF_8 Gy_‐EVs and CAF_8 Gy_‐CM according to qRT‐PCR. (F) TE‐1 and KYSE150 cells were treated with CM from CAF_8 Gy_ without EV depletion or with EV depletion by ultracentrifugation; the expression levels of miR‐193a‐3p in TE‐1 and KYSE150 cells were determined using qRT‐PCR. (G) TE‐1 and KYSE150 cells were treated with CM from CAF_8 Gy_ without EV depletion or with EV depletion using GW4869; the expression levels of miR‐193a‐3p in TE‐1 and KYSE150 cells were determined using qRT‐PCR. (H) CAF_8 Gy_‐CM was treated with RNase A (2 mg/mL) alone or in combination with Triton X‐100 (.1%) for 30 min, the expression levels of miR‐193a‐3p in CAF_8 Gy_‐CM were determined using qRT‐PCR. Data are presented as the mean ±  standard deviation (SD). ns, not significant, *p < .05, **p < .01, ***p < .001.

Exosomal regulatory activity is largely mediated by encapsulated miRNAs.^36^ miRNA profiling revealed significant differential expression in CAF_8 Gy_‐EVs compared to CAF_0 Gy_‐EVs, with miR‐193a‐3p and miR‐197‐3p being the most upregulated (Figure 2A). Quantitative PCR analyses of independent samples confirmed that miR‐193a‐3p exhibited a >10‐fold increase in CAF_8 Gy_‐EVs compared to CAF_0 Gy_‐EVs (Figure 2B). The intracellular level of miR‐193a‐3p was significantly increased by radiation in CAFs but not in ESCC cells (Figure 2C,D). Notably, treatment of ESCC cells with CAF_8 Gy_‐EVs led to a significant elevation of intracellular miR‐193a‐3p expression in ESCC cells (Figure 2E), suggesting that miR‐193a‐3p can be transferred from irradiated CAFs to ESCC cells. Similar results were obtained from ESCC cells treated with CM from irradiated CAF (Figure 2E). In CAF_8 Gy_‐CM‐treated ESCC cells, depletion of EVs in CAF‐derived CM could significantly decreased the miR‐193a‐3p levels in cancer cells (Figure 2F,G). Moreover, treating the CAF_8 Gy_‐CM with RNase and Triton X‐100 led to a significantly reduced miR‐193a‐3p level in CM‐treated ESCC cells compared to treatment with RNase alone (Figure 2H), suggesting that EV mediates the intercellular transfer of miR‐193a‐3p from irradiated CAFs to ESCC cells. Previous studies have shown that radiation activates multiple signalling pathways, including the MEK/ERK and TGF‐β/Smad pathways, both of which are involved in miRNA biogenesis.^37^, ^38^, ^39^, ^40^, ^41^ Our results revealed that treatment with SB431542, a TGF‐β/Smad pathway inhibitor, completely abolished the irradiation‐induced upregulation of miR‐193a‐3p, while U0126, a MEK/ERK pathway inhibitor, had no effect (Supporting Information Figure 4). These results suggest that the TGF‐β/Smad pathway plays a key role in mediating the irradiation‐induced upregulation of miR‐193a‐3p in CAFs.

### 3.3 Irradiated CAF‐derived EV miR‐193a‐3p promotes the metastasis and EMT of ESCC cells

**FIGURE 3:**
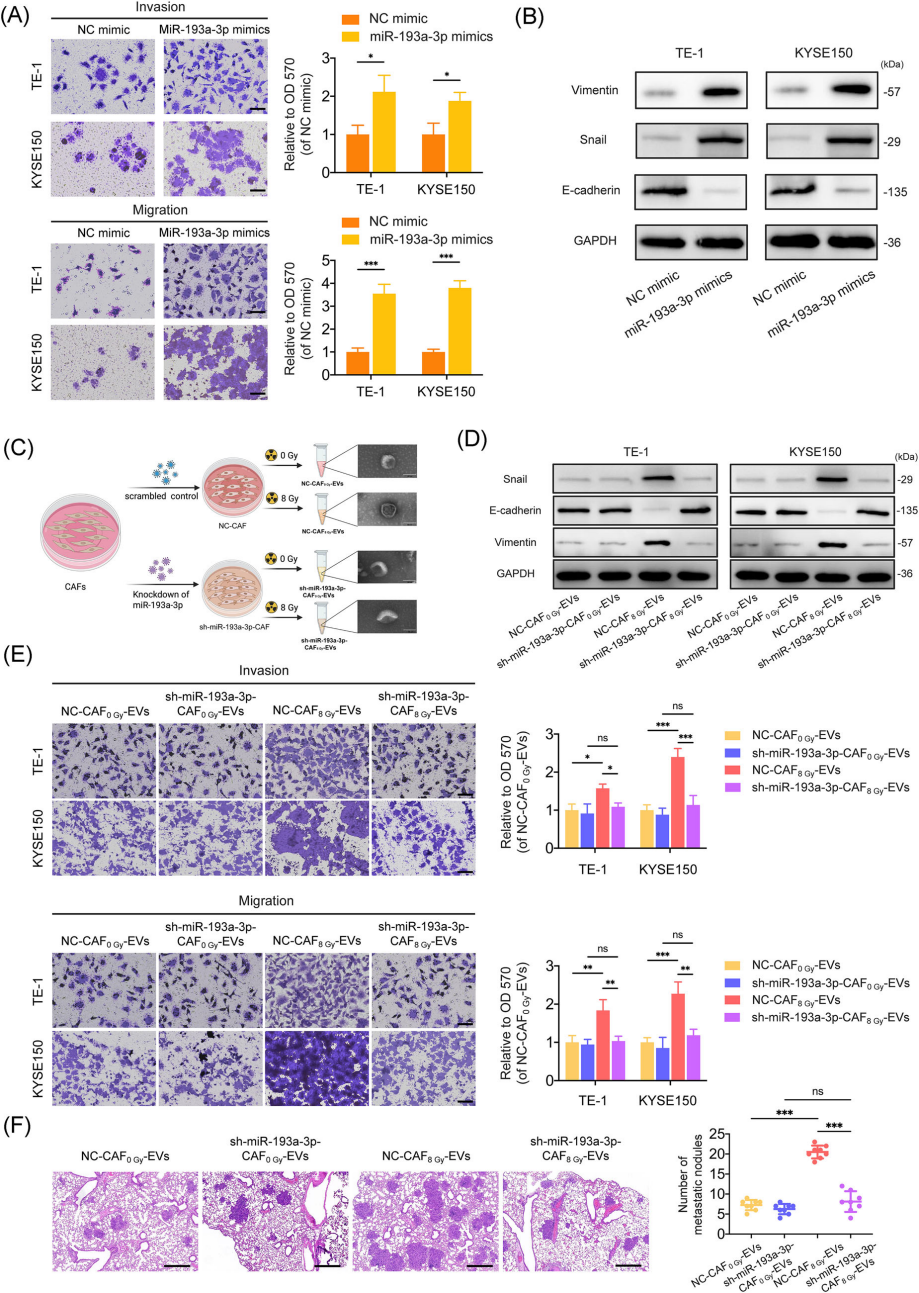
Radiation‐induced release of extracellular vesicle (EV) miR‐193a‐3p from cancer‐associated fibroblasts (CAFs) promoted the metastasis and epithelial–mesenchymal transition (EMT) of oesophageal squamous cell cancer (ESCC) cells. (A) Effects of miR‐193a‐3p mimics on the invasion and migration abilities of TE‐1 and KYSE150 cells according to Transwell assay (crystal violet staining). The absorbance of eluted crystal violet was read at 570 nm. Scale bar: 50 µm. (B) Western blot analysis for EMT markers (Snail, Vimentin and E‐cadherin) in TE‐1 and KYSE150 cells transfected with NC mimic and miR‐193a‐3p mimics. (C) Schematic diagram of the method for obtaining NC‐CAF_0 Gy_‐EVs, sh‐miR‐193a‐3p‐CAF_0 Gy_‐EVs, NC‐CAF_8 Gy_‐EVs and sh‐miR‐193a‐3p‐CAF_8 Gy_‐EVs. (D) Western blot analysis for EMT markers (Snail, Vimentin and E‐cadherin) in TE‐1 and KYSE150 cells treated with NC‐CAF_0 Gy_‐EVs, sh‐miR‐193a‐3p‐CAF_0 Gy_‐EVs, NC‐CAF_8 Gy_‐EVs or sh‐miR‐193a‐3p‐CAF_8 Gy_‐EVs. (E) Effects of NC‐CAF_0 Gy_‐EVs, sh‐miR‐193a‐3p‐CAF_0 Gy_‐EVs, NC‐CAF_8 Gy_‐EVs and sh‐miR‐193a‐3p‐CAF_8 Gy_‐EVs on the invasion and migration abilities of TE‐1 and KYSE150 cells according to Transwell assay (crystal violet staining). The absorbance of eluted crystal violet was read at 570 nm. Scale bar: 50 µm. (F) Effects of NC‐CAF_0 Gy_‐EVs, sh‐miR‐193a‐3p‐CAF_0 Gy_‐EVs, NC‐CAF_8 Gy_‐EVs and sh‐miR‐193a‐3p‐CAF_8 Gy_‐EVs on the formation of metastatic lung nodules (*n* = 8 mice per group). Representative haematoxylin–eosin (H&E) staining photographs of metastatic lung nodules are shown, and the numbers of metastatic lung nodules were calculated. Scale bar: 500 µm. Data are presented as the mean ± standard deviation (SD). *p < .05, **p < .01, ***p < .001.

To investigate the functional role of miR‐193a‐3p, we overexpressed miR‐193a‐3p in ESCC cells via transfection with miR‐193a‐3p mimics (Supporting Information Figure 2A). It was shown that miR‐193a‐3p mimics exhibited markedly enhanced the migration and invasion abilities of ESCC cells (Figure 3A). Meanwhile, ESCC cells transfected with miR‐193a‐3p mimics exhibited a higher expression of Vimentin and Snail and lower E‐cadherin expression (Figure 3B). To assess whether miR‐193a‐3p mediates the pro‐metastatic effects of EVs derived from irradiated CAFs, we knocked down miR‐193a‐3p in CAFs and then exposed the cells to 0 Gy or 8 Gy of X‐ray radiation (Figure 3C). qRT‐PCR confirmed the significantly decreased levels of miR‐193a‐3p in both miR‐193a‐3p‐knockdowned CAFs and CAF‐derived EVs (Supporting Information Figure 2B). ESCC cells treated with EVs from irradiated CAFs with miR‐193a‐3p knockdown failed to promote ESCC cell invasion, migration or EMT (Figure 3D,E). Consistent findings were also observed in vivo, where EVs from irradiated CAFs with miR‐193a‐3p knockdown failed to promote ESCC cell metastasis or EMT (Figure 3F and Supporting Information Figure 2C), suggesting that miR‐193a‐39 mediates the pro‐metastatic role of irradiated CAF‐derived EV.

### 3.4 miR‐193a‐3p promotes ESCC metastasis through PTEN/Akt‐mediated EMT

**FIGURE 4:**
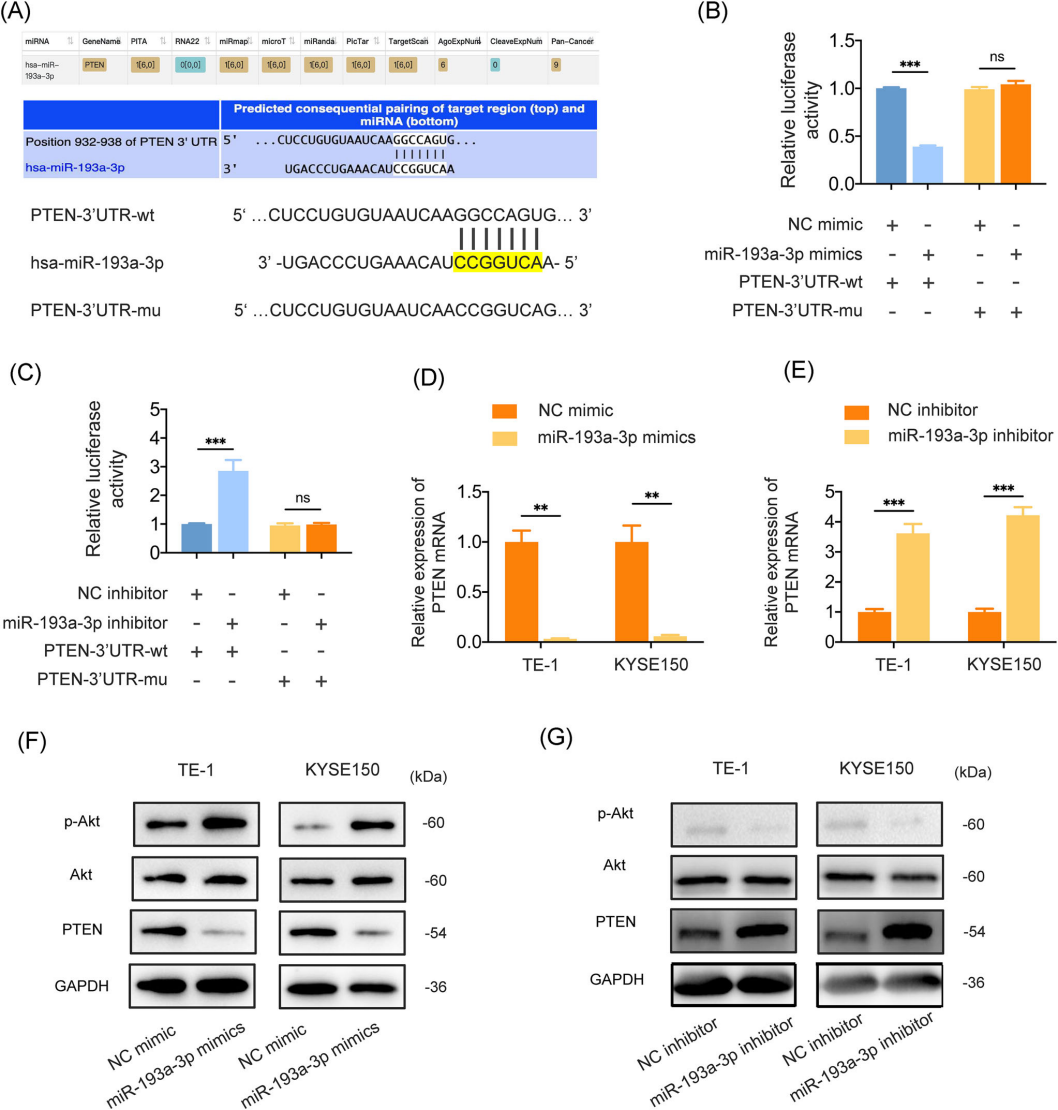
PTEN was a direct target of miR‐193a‐3p. (A) The putative miR‐193a‐3p complementary site in the 3′UTR of PTEN (PTEN 3′UTR‐wt) and the corresponding mutated sequence within the predicted miR‐193a‐3p targeting regions (PTEN 3′UTR‐mu). Seed sequences are marked in yellow. (B) HEK293T cells were cotransfected with luciferase plasmids containing either wild‐type or mutant PTEN 3′UTR and miR‐193a‐3p mimics or NC mimic. The relative luciferase levels were assessed using a dual‐luciferase assay kit. (C) HEK293T cells were cotransfected with luciferase plasmids containing either wild‐type or mutant PTEN 3′UTR and miR‐193a‐3p inhibitor or NC inhibitor. The relative luciferase levels were assessed using a dual‐luciferase assay kit. (D) Relative expression of PTEN mRNA in TE‐1 and KYSE150 cells transfected with miR‐193a‐3p mimics or NC mimic according to quantitative reverse transcription polymerase chain reaction (qRT‐PCR). (E) Relative expression of PTEN mRNA in TE‐1 and KYSE150 cells transfected with miR‐193a‐3p inhibitor or NC inhibitor according to qRT‐PCR. (F) Western blot analysis for PTEN, Akt and p‐Akt in TE‐1 and KYSE150 cells transfected with miR‐193a‐3p mimics or NC mimic. (G) Western blot analysis for PTEN, Akt and p‐Akt in TE‐1 and KYSE150 cells transfected with miR‐193a‐3p inhibitor or NC inhibitor. Data are presented as the mean ± standard deviation (SD). *p < .05, **p < .01, ***p < .001.

To elucidate the downstream molecular mechanisms of miR‐193a‐3p, we used the starBase website (http://starbase.sysu.edu.cn/)^42^ to identify the target of miR‐193a‐3p. Selection criteria included identification by five prediction programs (miRmap, microT, miRanda, PicTar and TargetScan). PTEN was identified as one of the potential target genes, and the ‘seed sequence’ of miR‐193a‐3p matched the 3′ untranslated region (3′UTR) of PTEN (Figure 4A). Our luciferase reporter assay confirm the targeting relationship between miR‐193a‐3p and PTEN. miR‐193a‐3p mimics significantly suppressed, whereas miR‐193a‐3p inhibitors significantly enhanced the luciferase activity in cells transfected with a luciferase reporter vector containing wild‐type PTEN 3′‐UTR, but not in cells transfected with vector containing mutant PTEN 3′‐UTR (Figure 4B,C). Previous studies have demonstrated that miRNAs regulate gene expression by binding to the 3′UTR s of target mRNAs, leading to their degradation.^43^, ^44^, ^45^ Based on this, we hypothesised that miR‐193a‐3p could influence PTEN mRNA stability and therefore conducted PTEN mRNA decay assays. Our results showed that miR‐193a‐3p overexpression significantly accelerated PTEN mRNA degradation, suggesting that miR‐193a‐3p binding to the PTEN 3′UTR promotes its degradation at the post‐transcriptional level (Supporting Information Figure 3).

**FIGURE 5:**
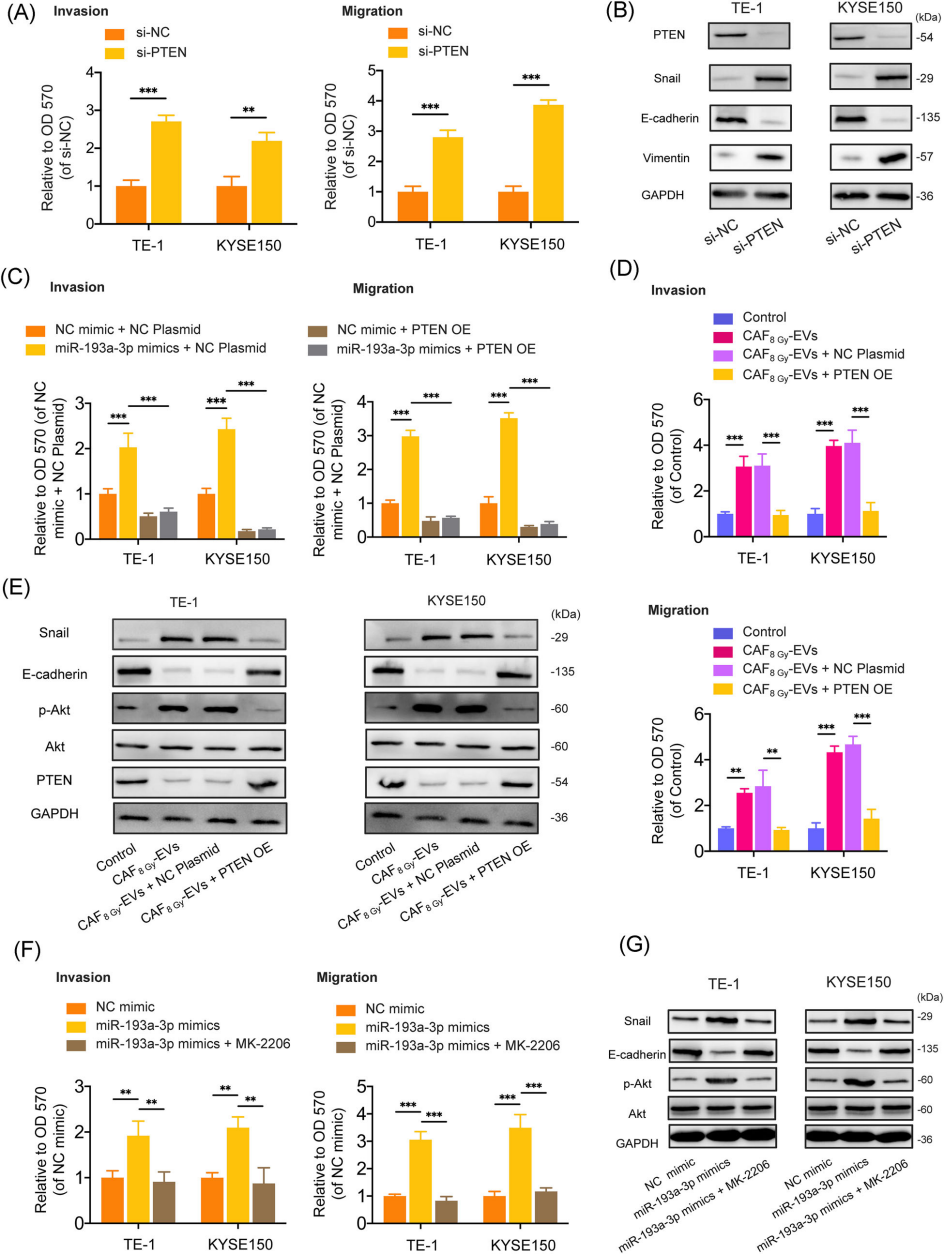
miR‐193a‐3p promoted the epithelial–mesenchymal transition (EMT) and metastasis of oesophageal squamous cell cancer (ESCC) by inhibiting PTEN and activating the Akt signalling pathway. (A) TE‐1 and KYSE150 cells were transfected with PTEN small interfering RNA (si‐PTEN) or the corresponding control (si‐NC) and then harvested for the Transwell assay. The absorbance of eluted crystal violet was read at 570 nm. (B) Western blot analysis for PTEN and EMT markers (Snail, Vimentin and E‐cadherin) in TE‐1 and KYSE150 cells transfected with si‐PTEN or si‐NC. (C) TE‐1 and KYSE150 cells were transfected with miR‐193a‐3p mimics and plasmids of PTEN (PTEN OE) or the corresponding control (NC plasmid) and then harvested for the Transwell assay. The absorbance of eluted crystal violet was read at 570 nm. (D) TE‐1 and KYSE150 cells were transfected with PTEN OE or NC plasmid and treated with CAF_8 Gy_‐extracellular vesicles (EVs). Cells were harvested for the Transwell assay. The absorbance of eluted crystal violet was read at 570 nm. (E) TE‐1 and KYSE150 cells were transfected with PTEN OE or NC plasmid and treated with CAF_8 Gy_‐EVs. The protein levels of PTEN, Akt, p‐Akt, E‐cadherin and Snail were assessed by Western blot analysis. (F) TE‐1 and KYSE150 cells were transfected with miR‐193a‐3p mimics and then treated with or without MK‐2206 (10 µM). Cells were harvested for the Transwell assay. The absorbance of eluted crystal violet was read at 570 nm. (G) TE‐1 and KYSE150 cells were transfected with miR‐193a‐3p mimics and then treated with or without MK‐2206 (10 µM). The protein levels of Akt, p‐Akt, E‐cadherin and Snail were assessed via Western blot analysis. Data are presented as the mean ± standard deviation (SD). *p < .05, **p < .01, ***p < .001.

PTEN is known as a negative regulator of Akt,^46^ which plays a critical role in EMT and cancer metastasis.^47^ As expected, miR‐193a‐3p overexpression reduced PTEN expression and increased phosphorylated Akt (p‐Akt) levels in ESCC cells (Figure 4D,F), while miR‐193a‐3p inhibited increased PTEN expression and decreased p‐Akt levels in ESCC cells (Figure 4E,G). To characterise the role of PTEN in miR‐193a‐3p‐mediated ESCC progression, ESCC cells were transfected with siRNAs for PTEN. PTEN knockdown significantly enhanced invasion, migration and EMT of ESCC cells (Figure 5A,B and Supporting Information Figure 5A). Conversely, PTEN overexpression attenuated the pro‐migratory and pro‐invasive effects of miR‐193a‐3p mimics (Figure 5C and Supporting Information Figure 5B) and CAF_8 Gy_‐EVs (Figure 5D and Supporting Information Figure 5C). ESCC cells treated with CAF_8 Gy_‐EVs exhibited downregulated PTEN and E‐cadherin expression, along with upregulated p‐Akt and Snail expression. However, PTEN overexpression reversed the regulatory effects of CAF_8 Gy_‐EVs on EMT‐related proteins (Figure 5E). Next, we investigated whether EV miR‐193a‐3p promotes ESCC invasion and migration via the Akt signalling pathway. As shown, Akt inhibitor (MK‐2206) significantly attenuated miR‐193a‐3p‐induced invasion and migration of ESCC cells (Figure 5F and Supporting Information Figure 6A). MK‐2206 also abolished miR‐193a‐3p‐mediated E‐cadherin downregulation and Snail upregulation in ESCC cells (Figure 5G). Similarly, MK‐2206 attenuated the effects of CAF_8 Gy_‐EVs on the invasion and migration abilities and the expressions of Snail and E‐cadherin in ESCC cells (Supporting Information Figure 6B,C). Together, these results suggest that irradiated CAF‐derived EV miR‐193a‐3p promotes ESCC metastasis through PTEN/Akt pathway.

### 3.5 CAF‐derived miR‐193a‐3p–PTEN–Akt axis regulates ESCC metastasis and EMT in vivo after local radiotherapy

**FIGURE 6:**
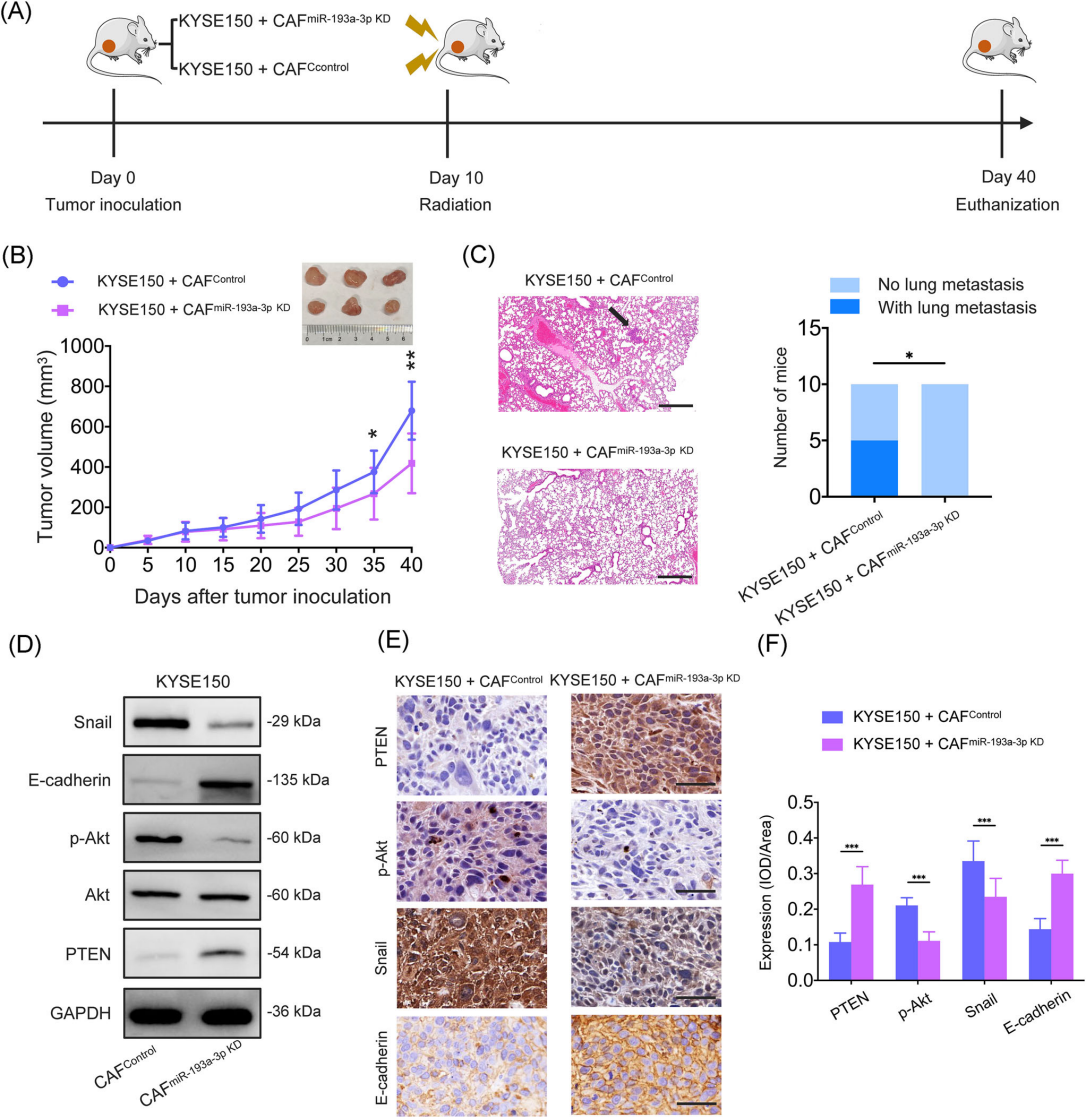
In vivo role of the cancer‐associated fibroblast (CAF)‐derived miR‐193a‐3p–PTEN–Akt axis in regulating oesophageal squamous cell cancer (ESCC) metastasis and epithelial–mesenchymal transition (EMT) after local radiotherapy. (A) Schematic diagram of animal treatment and timeline. Mixed cells (KYSE150 + CAF^Control^; KYSE150 + CAF^miR‐193a‐3p KD^) were subcutaneously inoculated into the right flank area of 5‐week‐old nude mice (*n* = 10 mice per group). (B) Tumour volumes were recorded and calculated. The tumours were excised on the 40th day after inoculation. Representative tumour images in the KYSE150 + CAF^Control^ and KYSE150 + CAF^miR‐193a‐3p KD^ groups are shown. (C) Lung metastasis formation was followed on the 40th day after inoculation. Representative haematoxylin–eosin (H&E) staining images of metastatic lung nodules in the KYSE150 + CAF^Control^ and KYSE150 + CAF^miR‐193a‐3p KD^ groups are shown (left), and lung metastasis in the KYSE150 + CAF^Control^ and KYSE150 + CAF^miR‐193a‐3p KD^ groups was analysed with the Fisher's exact test (right). Scale bar: 500 µm. (D) The protein levels of PTEN, Akt, p‐Akt, E‐cadherin and Snail in the xenograft tumour tissues were assessed via Western blot analysis. (E and F) Immunohistochemistry analysis of PTEN, p‐Akt, Snail and E‐cadherin in the xenograft tumour tissues. Scale bar: 50 µm. The images were analysed using ImageJ software by calculating the integrated optical density (IOD) per stained area. Data are presented as the mean ± standard deviation (SD) from 10 mice per group. *p < .05, **p < .01, ***p < .001.

To investigate the in vivo role of the miR‐193a‐3p–PTEN–Akt axis in regulating ESCC metastasis after local radiotherapy, we constructed CAFs in which miR‐193a‐3p was knocked down (CAF^miR‐193a‐3p KD^). KYSE150 and CAF^miR‐193a‐3p KD^ were subcutaneously coinjected into the right flank of nude mice to establish xenograft models. Tumours were locally irradiated with a single dose of 6 Gy at day 10 post‐inoculation (Figure 6A). We used a 6 Gy radiation dose in vivo, inducing miR‐193a‐3p upregulation comparable to that of an 8 Gy dose (Supporting Information Figure 7). Tumour volumes did not differ significantly between the KYSE150 + CAF^Control^ and KYSE150 + CAF^miR‐193a‐3p KD^ groups at day 10 after inoculation. However, by day 40, tumours in the KYSE150 + CAF^miR‐193a‐3p KD^ group were smaller than those in the KYSE150 + CAF^Control^ group (Figure 6B). Additionally, lung metastases were observed in five of 10 mice in the KYSE150+ CAF^Control^ group, whereas none of the 10 mice in the KYSE150 + CAF^miR‐193a‐3p KD^ group exhibited lung metastases (5/10 vs. 0/10; *p* < .05; Figure 6C). Immunohistochemistry and Western blotting revealed that xenograft tumours in the KYSE150 + CAF^miR‐193a‐3p KD^ group exhibited increased levels of PTEN and E‐cadherin and decreased p‐Akt and Snail expression compared with the KYSE150 + CAF^Control^ group (Figure 6D,F). These in vivo data further support the contribution of miR‐193a‐3p–PTEN–Akt axis to ESCC recurrence and metastasis after radiotherapy.

### 3.6 Upregulation of miR‐193a‐3p correlates with poor prognosis in patients with ESCC

**FIGURE 7:**
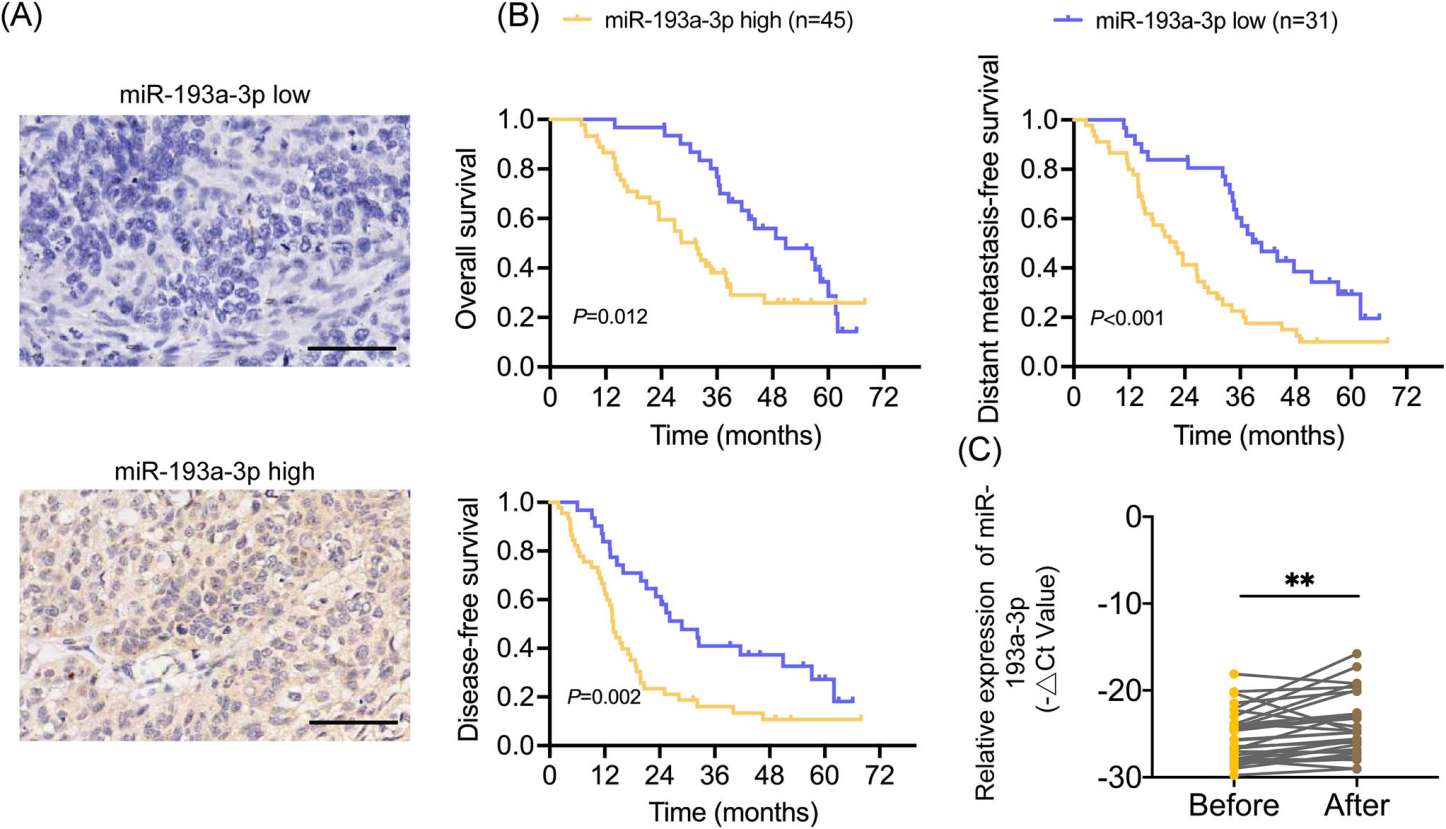
Upregulation of miR‐193a‐3p was associated with the poor prognosis of patients with oesophageal squamous cell cancer (ESCC). (A) Low expression of miR‐193a‐3p and high expression of miR‐193a‐3p in ESCC tissues as indicated by in situ hybridisation. Scale bar: 100 µm. (B) Kaplan–Meier analysis of overall survival, distant metastasis‐free survival and disease‐free survival in patients with ESCC from the high‐miR‐193a‐3p and low‐miR‐193a‐3p groups. (C) Extracellular vesicle (EV) miR‐193a‐3p in the plasma of patients with ESCC at 1 day before radiotherapy and at 14 days after the initiation of radiotherapy. Cel‐miR‐39 was used as an external control for EV miRNA detection. **p < .01.

To investigate the clinical significance of miR‐193a‐3p, in situ hybridisation was performed on tumour samples from a retrospective cohort of 76 patients with ESCC. Based on miR‐193a‐3p expression levels, patients were classified into miR‐193a‐3p‐high and miR‐193a‐3p‐low groups (Figure 7A). The associations between miR‐193a‐3p expression and the clinicopathological characteristics are summarised in Supporting Information Table 4. High miR‐193a‐3p expression was significantly associated with the advanced T stage, advanced N stage and advanced TNM stage. Kaplan–Meier survival curves demonstrated that patients in the miR‐193a‐3p‐high group had significantly shorter DFS, DMFS and OS compared with those in the miR‐193a‐3p‐low group (Figure 7B). Univariate and multivariate Cox regression analyses identified miR‐193a‐3p as an independent predictor of poor prognosis in patients with ESCC (Table 1 and Supporting Information Tables 5 and 6). To further investigate the radiotherapy‐induced changes in miR‐193a‐3p levels, we analysed the miR‐193a‐3p expression in plasma EVs from a prospective cohort of 32 patients undergoing concurrent definitive chemoradiation therapy. Plasma samples were collected 1 day before radiotherapy and 14 days after its initiation. The radiotherapy regimen is summarised in Supporting Information Table 7. Notably, EV miR‐193a‐3p levels were significantly elevated following radiotherapy (Figure 7C). Together, these clinical data suggest that radiotherapy induces the expression of miR‐193a‐3p, which results in unfavourable outcomes in patients with ESCC.

**TABLE 1:** Univariate and multivariate Cox proportional hazards model for overall survival (OS) in patients with ESCC (*n* = 76).

	Univariate analysis		Multivariate analysis	
Variable	HR (95% CI)	*p*	HR (95% CI)	*p*
Age, year (≤60 vs. >60)	.727 (.415–1.275)	.267		
Sex (male vs. female)	.858 (.440–1.674)	.653		
Tumour location				
Lower	2.064 (.610–6.990)	.244		
Middle	2.203 (.670–7.242)	.193		
Upper	Ref	–		
Tumour differentiation				
Poor	2.270 (.942–5.469)	.068	3.241 (1.293–8.124)	.012
Moderate	1.735 (.751–4.008)	.197	1.592 (.688–3.682)	.278
Well	Ref	–	Ref	–
Pathologic T stage (T1 + T2 vs. T3 + T4)	.476 (.266–.853)	.013	.747 (.378–1.478)	.402
Tumour length, cm (<5 vs. ≥5)	1.252 (.624–2.512)	.528		
Pathologic N stage (N0 + N1 vs. N2 + N3)	.262 (.146–.472)	<.001	.307 (.154–.610)	<.001
Adjuvant therapy (Yes vs. No)	1.468 (.839–2.566)	.179		
miR‐193a‐3p expression (low vs. high)	.488 (.276–.864)	.014	.496 (.265–.929)	.028

Abbreviations: CI, confidence interval; HR, hazard ratio.

## 4 DISCUSSION

Radiotherapy remains a cornerstone in the management of inoperable or locally advanced ESCC.^48^ However, despite its clinical efficacy, distant recurrence remains a significant barrier to long‐term tumour control. Understanding the underlying mechanisms driving tumour metastasis after radiotherapy is crucial for improving patient prognosis.

The interaction between cancer cells and surrounding stromal components, such as CAFs, is actively studied, with evidence suggesting that cellular communication between cancer cells and CAFs plays a vital role in tumour progression.^49^ Radiation‐induced stress alters the stromal microenvironment, leading to significant cellular and molecular changes. The presence of activated CAFs is correlated with poor prognosis after definitive chemoradiation for ESCC.^50^, ^51^ Additionally, radiation‐exposed fibroblasts demonstrate enhanced protumourigenic and promalignant properties.^52^, ^53^, ^54^, ^55^, ^56^, ^57^ However, few studies have examined cancer–CAF crosstalk in ESCC following radiotherapy.

In this study, we demonstrated that irradiated CAFs enhanced the invasion and migration of ESCC cells, consistent with Bao et al.^58^ Furthermore, we provide the first direct evidence that EVs mediate intercellular communication between irradiated CAFs and cancer cells, promoting ESCC metastasis. Radiation‐enhanced CAF pro‐malignant functions have also been observed in other cancer types. For example, Ohuchida et al. reported that irradiated CAFs promoted the invasiveness of pancreatic cancer cells in both in vitro coculture systems and in vivo orthotopic murine xenograft models.^53^ These effects may be attributed to upregulated c‐Met expression and increased MAPK activity in pancreatic cancer cells.^53^ Similarly, in coculture systems of pancreatic cancer cells with irradiated or non‐irradiated CAFs, Li et al. reported that irradiated CAFs enhanced pancreatic cancer cell invasiveness and EMT.^59^ Tommelein et al. found that irradiated CAFs promoted colorectal cancer progression via increased paracrine IGF1/IGF1R signalling.^23^ Our findings, along with these previous studies, suggest that combining radiotherapy with CAF‐targeted interventions may enhance the anti‐tumour effects of radiotherapy and improve patient outcomes. Furthermore, a larger target volume or increased scattered radiation exposure can activate CAFs, potentially worsening cancer prognosis. Therefore, careful monitoring of the CAF irradiation dose surrounding the target tumour volume is crucial.

Besides CAFs, radiotherapy also exerts dual effects on other key cells within the TME, influencing both anti‐tumour immunity and pro‐tumour phenotypes. On the one hand, radiotherapy promotes tumour cell antigen release, activating dendritic cells (DCs) that prime CD8⁺ T cells for anti‐tumour responses. It also enhances CD8⁺ T cell cytotoxicity by stimulating the secretion of TNF‐α, IFN‐γ and granzyme B.^60^ Furthermore, low‐dose radiation activates macrophages, polarising them into the M1 phenotype, which improves tumour phagocytosis and recruits additional immune cells through TNF‐α and IL‐12 secretion.^61^ On the other hand, radiotherapy also facilitates pro‐tumour processes. It enhances the recruitment of regulatory T cells (Tregs) to the TME, contributing to immune suppression and promoting radioresistance. High‐dose radiation, in particular, induces macrophage polarisation towards the M2 phenotype,^62^ impairing phagocytosis and driving the secretion of TGF‐β, a key factor in tumour angiogenesis and immune evasion.

Regarding the mechanism by which irradiated CAFs enhance the invasion and migration of ESCC cells, we found that these CAFs release miR‐193a‐3p‐enriched EVs, which promote ESCC metastasis and EMT through PTEN downregulation and activation of the Akt signalling pathway. miR‐193a‐3p exerts diverse functions in multiple cancers. For example, studies suggest that miR‐193a‐3p functions as a tumour suppressor in colorectal,^63^ lung,^64^ and breast cancers,^65^ inducing apoptosis, suppressing cell proliferation and inhibiting invasion and migration. Conversely, miR‐193a‐3p acts as a tumour promoter in bladder cancer and promotes multichemoresistance.^66^ miR‐193a‐3p is upregulated in ESCC tissues,^67^, ^68^ and its high expression in ESCC tissues is positively correlated with the recurrence and poor prognosis of ESCC.^68^ In vitro analysis indicates that miR‐193a‐3p promotes ESCC proliferation, migration and radio‐ and chemo‐resistance.^67^, ^68^, ^69^ Our findings align with previous studies on the oncogenic role of miR‐193a‐3p in ESCC. To explore underlying mechanisms, we used bioinformatic analysis and identified PTEN as a potential target of miR‐193a‐3p. PTEN suppresses the PI3K/Akt pathway.^70^ Our previous study, involving immunohistochemical analysis of tissue microarrays from 275 patients with ESCC who underwent complete three‐field lymphadenectomy, showed that Akt1 activation correlates with poor prognosis.^71^ These findings suggest that miR‐193a‐3p or PI3K/Akt pathway could serve as potential therapeutic targets.

Our study shows that inhibiting miR‐193a‐3p in CAFs enhances radiotherapy efficacy, suggesting that miR‐193a‐3p inhibitors could complement cancer treatment. Significant progress has been made in developing miRNA inhibitors, such as antisense oligonucleotides (ASOs), which bind specifically to the target miRNA or its precursor to prevent their function, and RNA interference (RNAi) technologies, which utilise siRNAs or short hairpin RNAs (shRNAs) to silence miRNA expression.^72^, ^73^, ^74^ These strategies have demonstrated the potential to effectively block oncogenic miRNAs and reverse treatment resistance in cancer. Moreover, advances in RNA‐based delivery systems, such as lipid nanoparticles and viral vectors,^75^, ^76^ have facilitated the targeted delivery of these inhibitors to specific tissues, enhancing their therapeutic potential. Although our study were unable to specifically target miRNAs within EVs in our study. Despite this, targeting EV‐derived miRNAs remains a promising approach for improving the efficacy of radiotherapy in future research.

Liquid biopsy is an emerging tool for monitoring treatment response and predicting cancer outcomes.^77^ EVs, widely distributed in body fluids, dynamically reflect cancer progression and treatment resistance. For example, Liu et al. constructed a novel four‐serum exosomal miRNA nomogram as a predictive tool for preoperative lymph node metastasis in ESCC.^78^ Plasma exosomal miR‐340‐5p and serum exosomal miR‐339‐5p are associated with radiotherapy response and prognosis in ESCC.^79^, ^80^ In this study, we conducted a preliminary analysis of plasma EV miR‐193a‐3p levels before and after definitive radiotherapy in patients with ESCC. Plasma EV miR‐193a‐3p significantly increased after radiotherapy. Plasma EV miR‐193a‐3p shows potential as a dynamic prognostic biomarker after radiotherapy in ESCC, warranting further investigation. Compared to traditional biomarkers, miR‐193a‐3p offers clear advantages in the dynamic monitoring of treatment response and prognosis in cancer metastasis. Unlike static biomarkers, miR‐193a‐3p, carried by exosomes from irradiated CAFs, reflects real‐time changes in the TME.^81^ Its early elevation in plasma, observed as soon as 14 days post‐radiotherapy, allows for early detection of treatment effects. Specifically influenced by radiotherapy, miR‐193a‐3p enables precise differentiation of tumour‐related signals from other factors, enhancing its sensitivity and specificity. This makes it a promising biomarker for monitoring treatment response, recurrence and metastasis.

The metastatic impact of radiation‐induced EVs secreted by CAFs on ESCC cells may vary with radiation dose and fractionation schedules. In preliminary experiments, we examined how different fractionation regimens affected miR‐193a‐3p levels in CAF‐derived exosomes. It was shown that 8 Gy in four fractions produced effects comparable to a single 8‐Gy dose (Supporting Information Figure 8). A dose‐dependent relationship was observed between radiation exposure and miR‐193a‐3p levels in the exosomes. miR‐193a‐3p levels increased significantly with higher radiation doses. However, this relationship may not be strictly linear. Clinically, 60 Gy in 30 fractions is the standard regimen for definitive radiotherapy in oesophageal cancer.^82^, ^83^ Whether radiation exposure at the tumour site enhances CAF‐derived miR‐193a‐3p secretion warrants further clinical investigation.

Radiation has differential effects on various cell types within the ESCC TME. Therefore, it remains unclear whether the upregulation of miR‐193a‐3p specifically occurs in CAFs. According to the literature, tumour cells, macrophages, T cells, fibroblasts and epithelial cells constitute approximately 80% of the cellular components in the oesophageal cancer TME.^84^, ^85^ To investigate this, we performed qPCR to detect the expression level of miR‐193a‐3p in the human cell lines corresponding to the major cellular components of the ESCC microenvironment, both before and after irradiation. Our results indicate that miR‐193a‐3p upregulation occurred in both primary CAFs and normal MRC5 fibroblasts. In contrast, no upregulation of miR‐193a‐3p was observed in other cell types (Supporting Information Figure 9). To further investigate this, in our ongoing subsequent research, we will employ immunofluorescence combined with in situ hybridisation to confirm the specific upregulation of miR‐193a‐3p in CAFs within the tumour tissues of ESCC patients post‐radiotherapy.

In conclusion, we are the first to demonstrate that EVs derived from irradiated CAFs play a crucial role in intercellular communication, fostering a metastasis‐promoting microenvironment. Our findings support the notion that radiotherapy‐activated CAFs contribute to ESCC metastasis post‐radiotherapy and provide a rationale for targeting irradiated CAF‐driven miR‐193a‐3p‐Akt signalling to improve radiotherapy outcomes of ESCC patients.

## AUTHOR CONTRIBUTIONS

Yechun Pang, Tiantian Guo, Yue Zhou, Shanshan Jiang and Xi Yang designed and performed the experiments and analysed the data. Jianjiao Ni, Xiao Chu and Li Chu provided technical assistance for the animal studies. Yechun Pang, Tiantian Guo, Yue Zhou, Shanshan Jiang, Yida Li, Jianjiao Ni, Xiao Chu, Li Chu and Xi Yang collected and provided tumour tissues and plasma samples from patients with ESCC. Yechun Pang, Tiantian Guo, Yue Zhou, Shanshan Jiang and Xi Yang wrote and revised the manuscript. Zhengfei Zhu and Xi Yang developed the study concept, designed the experiments and supervised the study.

## CONFLICT OF INTEREST STATEMENT

The authors declare no conflicts of interest.

## ETHICS STATEMENT

This study was conducted in accordance with the Declaration of Helsinki and was approved by the Ethics Committee of Fudan University Shanghai Cancer Center (no. 0504323‐4‐2307E).

## CONSENT

Not applicable.

## Supporting information



Supporting Information

Supporting Information

## Data Availability

The data that support the findings of this study are available from the corresponding author upon reasonable request.
