# Supplementary material for: Radiation‐induced extracellular vesicles from cancer‐associated fibroblasts drive oesophageal squamous cell carcinoma metastasis via the miR‐193a‐3p/PTEN/Akt pathway
Source: Clin Transl Med. 2025 Sep 25;15(10):e70483. doi: 10.1002/ctm2.70483 (PMC12464349; doi:10.1002/ctm2.70483)
Supplement: Supplementary file 1 — Supporting Information [file CTM2-15-e70483-s001.pdf]

Supplementary Figure 1 EVs derived from irradiated CAFs promote the invasion and migration of ESCC cells

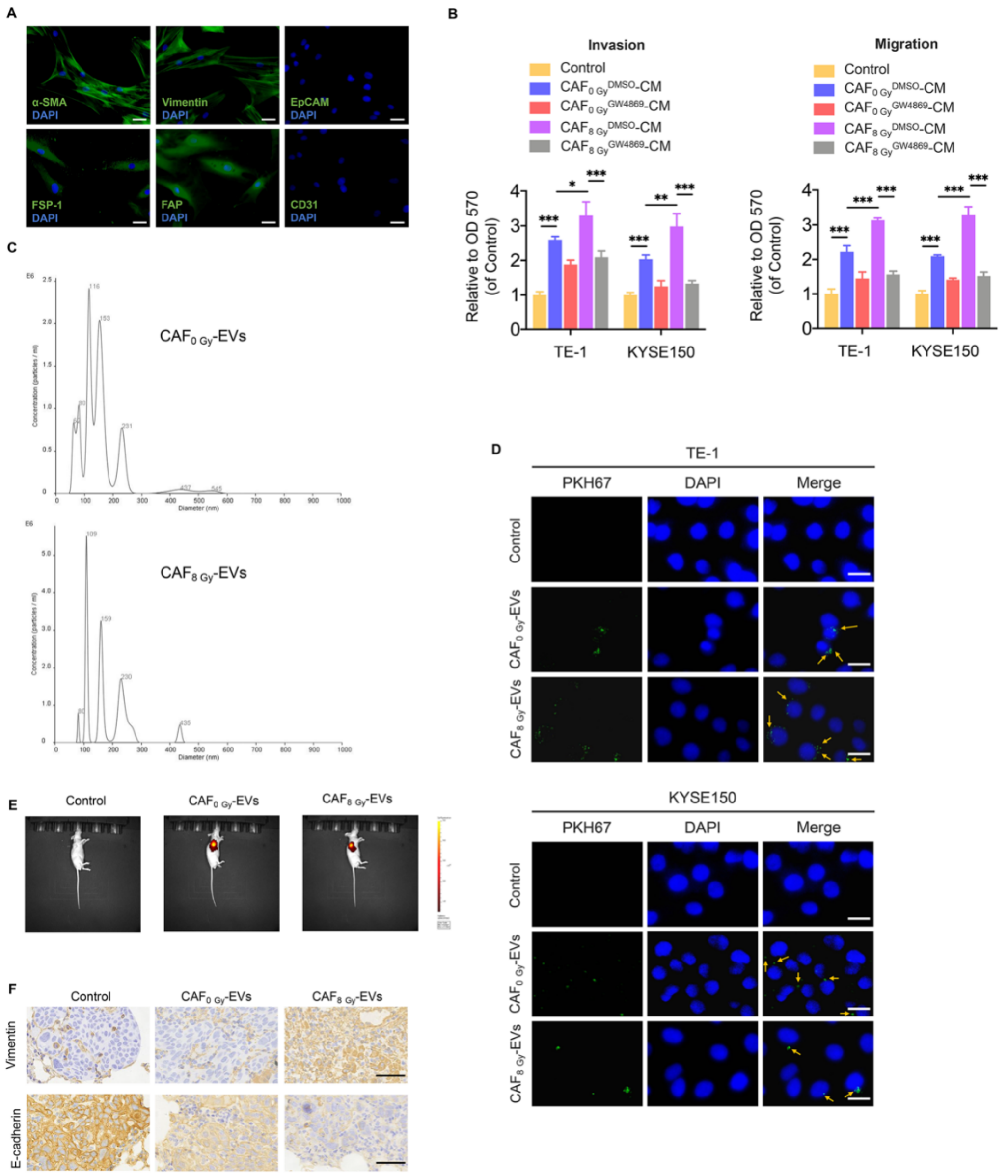

(A) Immunofluorescence staining for alpha-smooth muscle actin (α-SMA), fibroblast specific protein 1 (FSP-1), fibroblast activation protein (FAP), Vimentin, EpCAM, and CD31. Scale bar: 10 μm. (B) TE-1 and KYSE150 cells were treated with controls, conditioned medium (CM) from DMSO-treated $CAF_{0\ Gy}$, CM from GW4869-treated $CAF_{0\ Gy}$, CM from DMSO-treated $CAF_{8\ Gy}$, or CM from GW4869-treated $CAF_{8\ Gy}$ for 24 h. TE-1 and KYSE150 cells were then harvested for the Transwell assay (crystal violet staining). The absorbance of eluted crystal violet was read at 570 nm. (C) Nanosight tracking analysis (NTA) showing the size distribution

of isolated EVs. NTA revealed that the majority of EVs were distributed with a peak at size 116 nm for $CAF_{0\,Gy}$-EVs and 109 nm for $CAF_{8\,Gy}$-EVs. (D) Internalization of PKH67-labeled EVs (green) by TE-1 and KYSE150 cells observed under fluorescence microscopy. Scale bar: 20 μm. (E) In vivo fluorescence images of ESCC mouse xenograft model injected with DiR-labelled CAF-derived EVs at 48 h. (F) Immunohistochemistry for Vimentin and E-cadherin in the lung metastatic lesions. Scale bar: 50 μm. Data are presented as mean ± SD and are representative of three independent experiments. *$P < 0.05$, **$P < 0.01$, ***$P < 0.001$.

Supplementary Figure 2 Radiation-induced release of EV miR-193a-3p from CAFs promotes EMT of ESCC cells

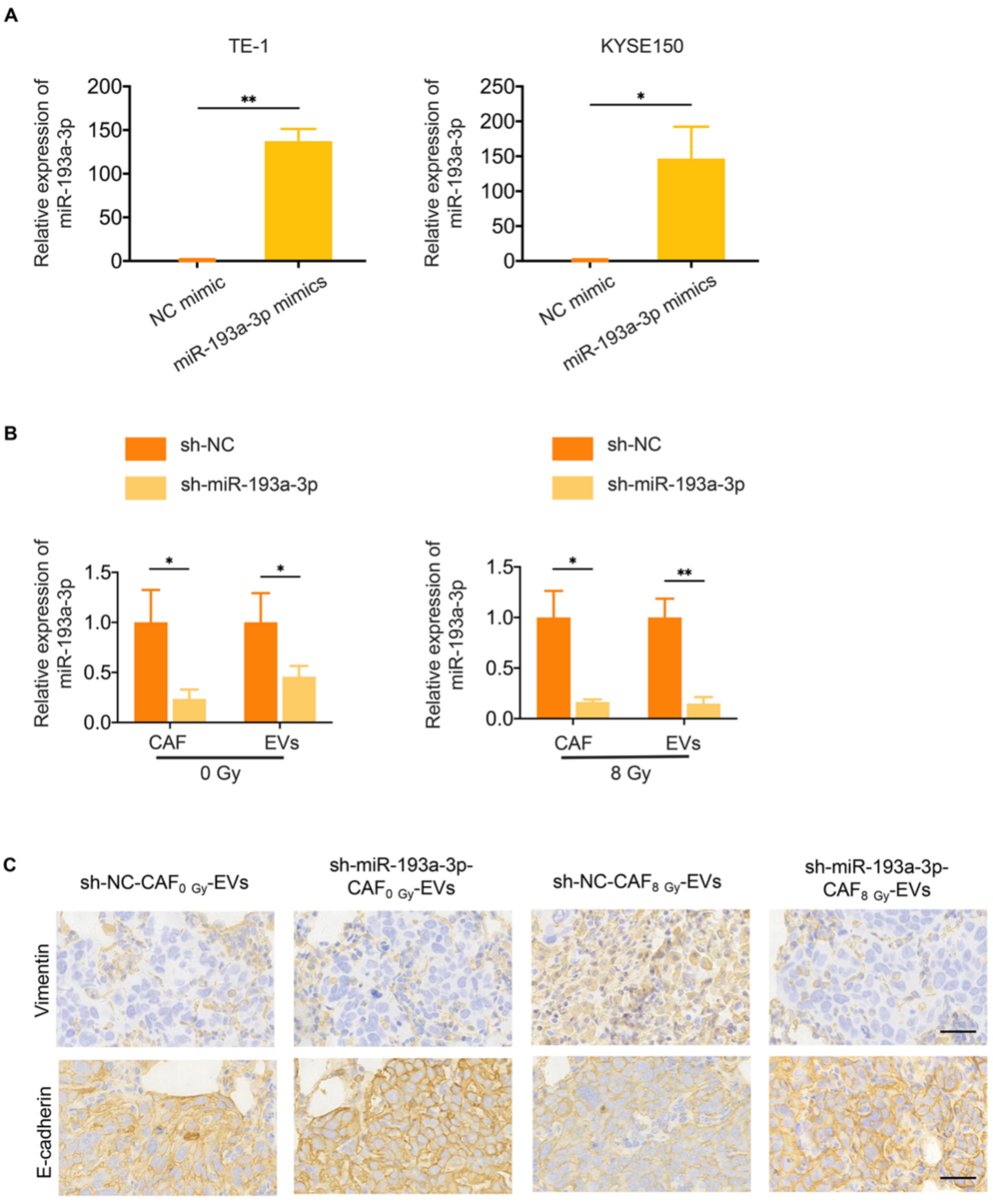

(A) Relative expression of miR-193a-3p in TE-1 and KYSE150 cells transfected with miR-193a-3p mimics or NC mimic. (B) CAFs were transduced with miR-193a-3p knockdown lentivirus and the corresponding controls, and then exposed to 0 Gy or 8 Gy of X-ray radiation. The miR-193a-3p expression levels in CAFs and CAF-derived EVs were determined using qRT-PCR. (C) Immunohistochemistry for Vimentin and E-cadherin in the lung metastatic lesions. Scale bar: 50 μm. Data are presented as mean ± SD and are representative of three independent experiments. $^*P < 0.05$, $^{**}P < 0.01$, $^{***}P < 0.001$.

Supplementary Figure 3 miR-193a-3p binding to the PTEN 3′UTR reduces PTEN expression by promoting mRNA degradation.

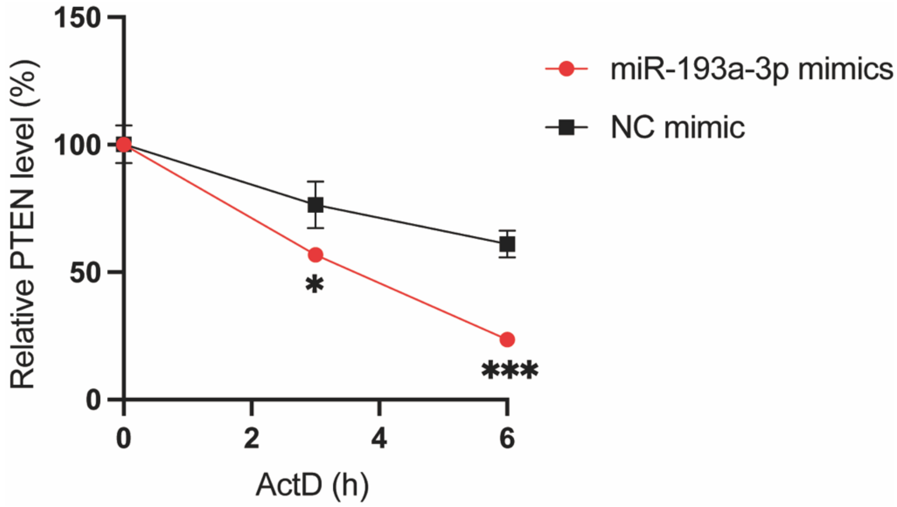

QPCR analysis of PTEN gene expression in TE-1 cells transfected with miR-193a-3p mimics or NC mimic at 0h, 3h, and 6h after Actinomycin D treatment. *$P < 0.05$, ***$P < 0.001$.

Supplementary Figure 4 TGF-β/Smad pathway is a key mechanism mediating the irradiation-induced upregulation of miR-193a-3p in CAFs

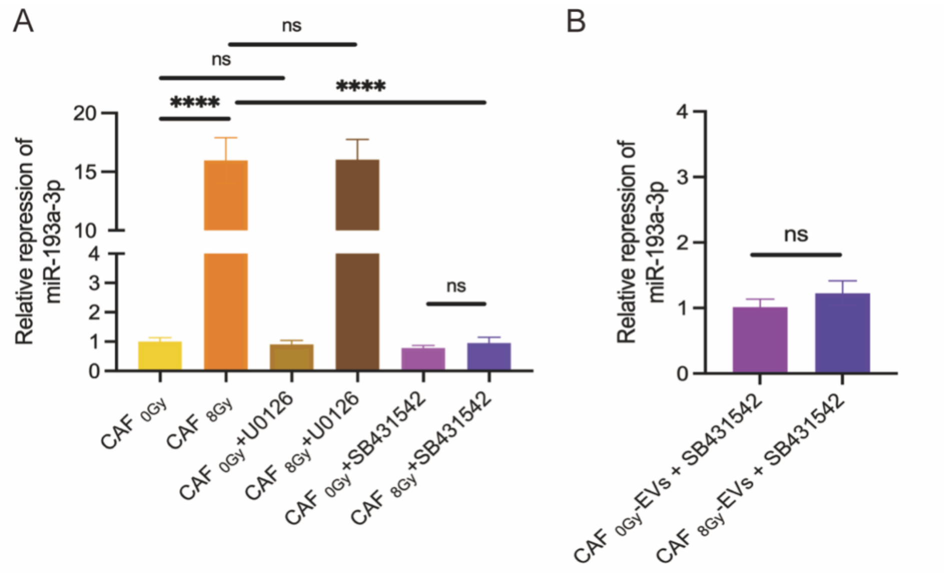

(A) QPCR analysis of miR-193a-3p expression levels in CAFs exposed to 0 Gy and 8 Gy, with or without treatment with U0126 (MEK/ERK pathway inhibitor) and SB431542 (TGF-β/Smad pathway inhibitor). (B) The expression levels of miR-193a-3p in EVs derived from CAFs exposed to 0 Gy and 8 Gy, both treated with SB431542, were analyzed by qPCR. Data are presented as mean ± SD and are representative of three independent experiments. ns, not significant; ****P < 0.0001.

Supplementary Figure 5 PTEN overexpression attenuated the promotive effects of miR-193a-3p on the invasion and migration abilities of ESCC cells

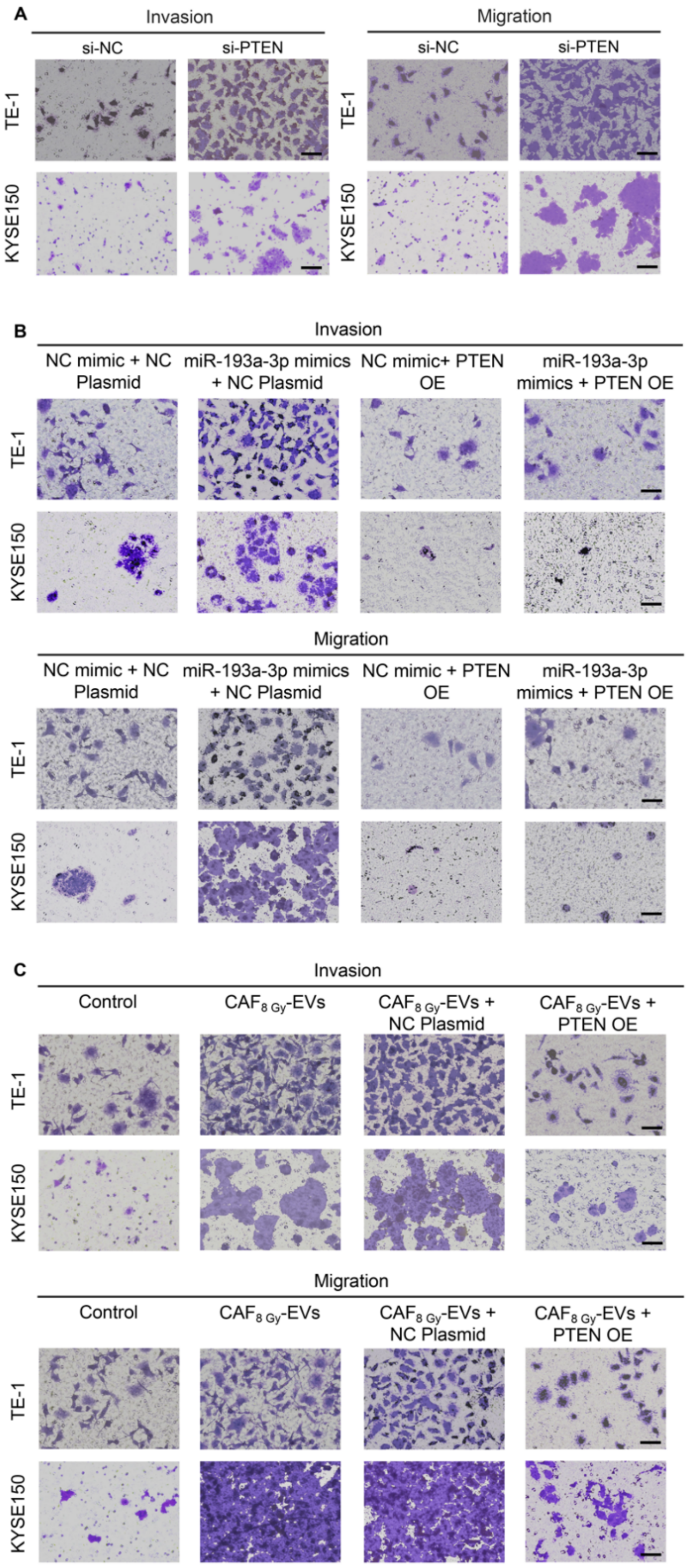

(A) Effects of si-PTEN on the invasion and migration abilities of TE-1 and KYSE150 cells by Transwell assay (crystal violet staining). TE-1 and KYSE150 cells were transfected with PTEN

small interfering RNA (si-PTEN) or the corresponding control (si-NC) and then harvested for the Transwell assay. Scale bar: 50 μm. (B) TE-1 and KYSE150 cells were transfected with miR-193a-3p mimics and plasmids of PTEN (PTEN OE) or the corresponding control (NC Plasmid). Cells were harvested for the Transwell assay. Scale bar: 50 μm. (C) TE-1 and KYSE150 cells were transfected with plasmids of PTEN (PTEN OE) or the corresponding control (NC Plasmid) and treated with $CAF_{8\ Gy}$-EVs. Cells were harvested for the Transwell assay. Scale bar: 50 μm.

Supplementary Figure 6 Akt inhibitor (MK-2206) attenuated the effects of radiation-induced EV miR-193a-3p from CAFs on the invasion and migration abilities of ESCC cells

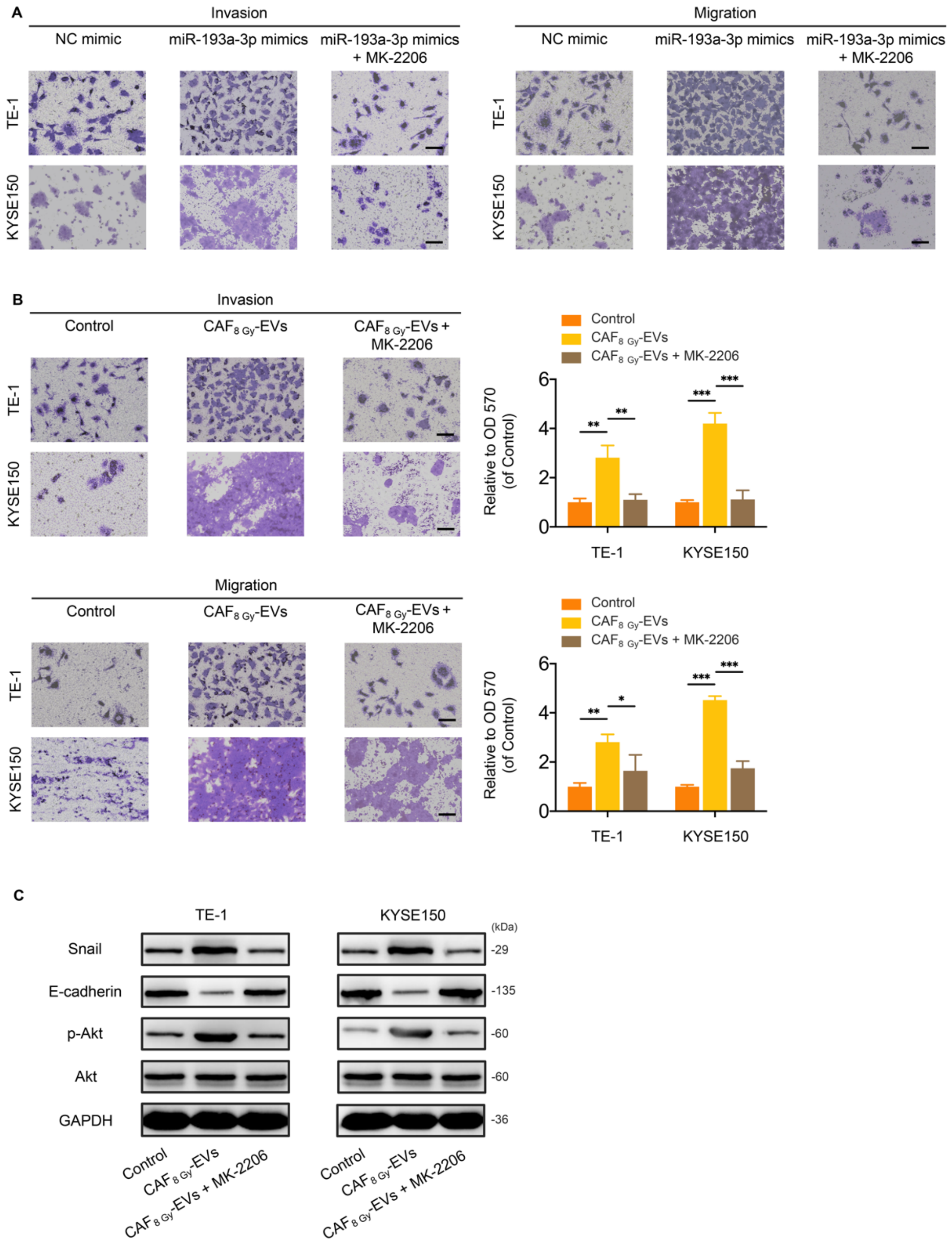

(A) TE-1 and KYSE150 cells were transfected with miR-193a-3p mimics and then treated with or without MK-2206 (10 μM). Cells were harvested for the Transwell assay. Scale bar: 50 μm. (B) TE-1 and KYSE150 cells were treated with $CAF_{8\ Gy}$-EVs and then treated with or without

MK-2206 (10 μM). Cells were harvested for the Transwell assay. The absorbance of eluted crystal violet was read at 570 nm. Scale bar: 50 μm. (C) TE-1 and KYSE150 cells were treated with $CAF_{8\ Gy}$-EVs and then treated with or without MK-2206 (10 μM). The protein levels of Akt, p-Akt, E-cadherin, and Snail were assessed by western blot analysis. Data are presented as mean ± SD. $*P < 0.05$, $**P < 0.01$, $***P < 0.001$.

Supplementary Figure 7. 6 Gy induces a miR-193a-3p response comparable to that of 8 Gy in CAFs

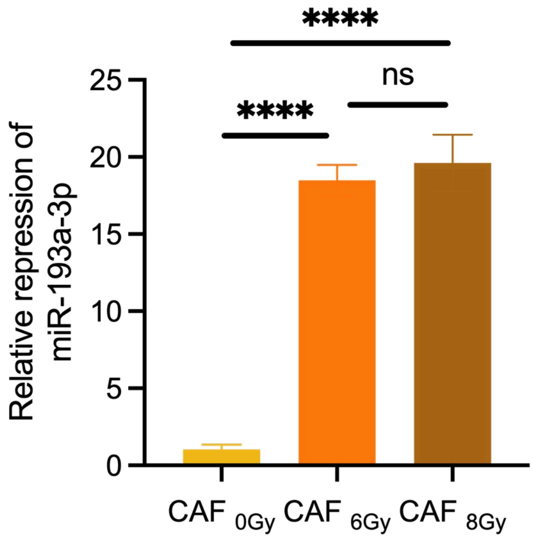

QPCR analysis of the expression levels of miR-193a-3p in CAFs exposed to 6 Gy and 8Gy. Data are presented as mean ± SD and are representative of three independent experiments. ns, not significant, ****P < 0.0001.

Supplementary Figure 8. 8 Gy in 4 fractions produced effects comparable to those of a single 8 Gy dose on radiation-induced release of EV miR-193a-3p

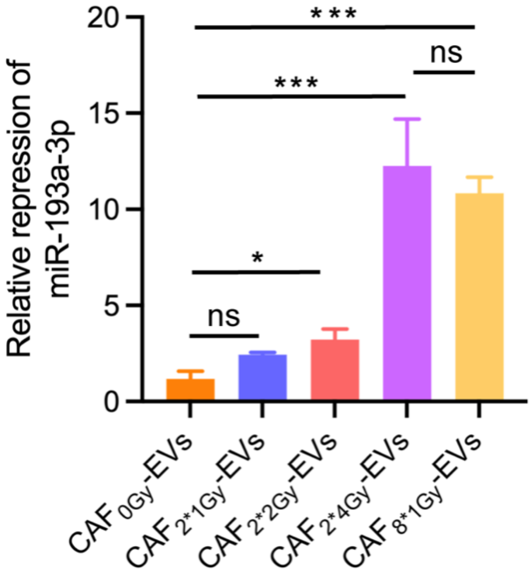

QPCR analysis of the expression levels of miR-193a-3p in EVs derived from CAFs exposed to different fractionated doses of X-rays (0 Gy, 2 * 1 Gy, 2 * 2 Gy, 2 * 4 Gy, 8 * 1 Gy). Data are presented as mean ± SD and are representative of three independent experiments. *P < 0.05, **P < 0.01, ***P < 0.001.

Supplementary Figure 9. Upregulation of miR-193a-3p specifically in fibroblasts within the ESCC tumor microenvironment

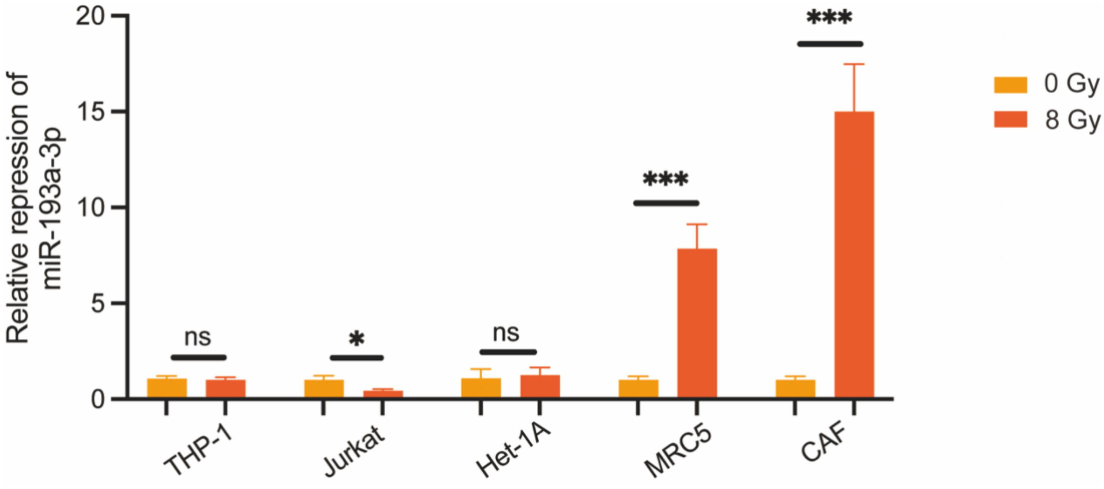

QPCR analysis of miR-193a-3p expression levels in various cells of the ESCC tumor microenvironment before and after 8 Gy irradiation. Data are presented as mean ± SD and are representative of three independent experiments. ns, not significant, $*P < 0.05$, $**P < 0.01$, $***P < 0.001$.
