# Supplementary material for: Radiation‐induced extracellular vesicles from cancer‐associated fibroblasts drive oesophageal squamous cell carcinoma metastasis via the miR‐193a‐3p/PTEN/Akt pathway
Source: Clin Transl Med. 2025 Sep 25;15(10):e70483. doi: 10.1002/ctm2.70483 (PMC12464349; doi:10.1002/ctm2.70483)
Supplement: Supplementary file 2 — Supporting Information [file CTM2-15-e70483-s002.pdf]

Supplementary Methods and Materials

Immunofluorescence

CAFs were fixed with 4% paraformaldehyde and then permeabilized with 0.2% Triton X-100 for 10 min. Afterwards, cells were blocked with 5% bovine serum albumin and treated with primary antibodies at 4 °C overnight, followed by incubation with Alexa Fluor 488-conjugated secondary antibody (Invitrogen; Thermo Fisher Scientific, Inc., Waltham, MA, USA) for 1 h at room temperature. Nuclei were counterstained with 4′,6-diamidino-2-phenylindole (DAPI; Beyotime, Shanghai, China). Cells were observed under fluorescence microscopy. The antibodies used for immunofluorescence are summarized in **Supplementary Table 2**.

PKH67-labeled EV internalization

Purified EVs derived from CAFs were labeled with PKH67 using PKH67 green fluorescent cell linker kits (Sigma-Aldrich, St. Louis, MO, USA) following the manufacturer’s protocol. PKH67-labeled EVs were co-cultured with ESCC cells at 37°C for 3 h. Afterward, cells were washed three times with PBS, followed by fixation with 4% paraformaldehyde for 30 min at room temperature. Cells were further stained with DAPI for 10 min to detect nuclei and visualized by fluorescence microscopy (Leica Microsystems, Wetzlar, Germany).

Blockade of EV secretion by GW4869

GW4869 (Sigma, USA) was dissolved in DMSO before dilution in CAF culture supernatant to achieve 20 μM GW4869 concentration in CAF culture condition. CAF were inoculated

into culture flasks. When CAF covers about 70% of the bottom of the culture flask, add GW4869 working solution (final concentration 20 μM) to the culture flask. After 24 h, the CAF was irradiated with 0 Gy or 8 Gy X-rays. After 24 h, the culture supernatant was collected.

***In vitro* migration and invasion assay**

Cell invasion assays were performed in 24-well transwell chamber (8.0-μm pore size, Millipore, Bedford, MA, USA) containing Matrigel (Corning). Cells (5×10^4 in 200 μL serum-free RPMI 1640 medium) were seeded into the upper chamber, and 600 μL RPMI 1640 with 10% FBS was added to the lower chamber. After 24 h incubation at 37 °C with 5% CO_2, the invaded cells in the lower chamber were fixed with 4% paraformaldehyde and stained with 0.1 % crystal violet. The stained crystal violet was then eluted using 33 % acetic acid, and absorbance of the eluted solution was read at 570 nm. A similar system, without coating with Matrigel, was used to assess cell migration *in vitro*.

Dual Luciferase activity assay

The putative miR-193a-3p complementary site in the 3'-UTR of PTEN (PTEN 3' UTR-WT) and the corresponding mutated sequence within the predicted miR-193a-3p targeting regions (PTEN 3' UTR-MUT) were inserted into the pSI-Check2-based luciferase reporter plasmids (Hanbio, Shanghai, China). Co-transfections of PTEN 3' UTR-WT or PTEN 3' UTR-MUT with miR-193a-3p mimics or mimic controls, and co-transfections of PTEN 3' UTR-WT or PTEN 3' UTR-MUT with miR-193a-3p inhibitor or inhibitor controls, into HEK 293T cells

were performed using Lipofectamine 3000 (Thermo Fisher Scientific). At 48 h after transfection, cells were collected, and the firefly and renilla luciferase activities were measured using the Dual-Luciferase Assay Kit (Promega, Madison, WI, USA). The firefly luciferase activity used for normalization.

RNAse A and Triton X-100 treatment

Conditioned medium from $CAF_{8\ Gy}$ was treated with RNase A (2 mg/ml) or RNase A (2 mg/ml) in combination with Triton X-100 (0.1%) for 20 min.

In situ hybridization

hsa-miR-193a-3p detection probe (5'-DIG-ACTGGGACTTTGTAGGCCAGTT-DIG-3') was used and DAB staining was performed according to manufacturer's protocol. Images were scanned using Pannoramic DESK, P-MIDI and assessed using CaseViewer software (3DHISTECH). The expression of miR-193a-3p in paraffin-embedded ESCC specimens was evaluated by two pathologists independently who were blinded to the clinical information of the patients. The staining score was generated by multiplying the proportion of positive cells with staining intensity. The proportion of positive cells was graded as follows: 0 (< 5%), 1 (6-25%), 2 (26-50%), 3 (51-75%), 4 (> 75%). The staining intensity was classified as follows: 0 (no staining), 1 (weak), 2 (moderate), and 3 (strong). Patients with a score greater than 3 were classified as the miR-193a-3p high group, while those with a score of 3 or lower were classified as the miR-193a-3p low group.

Immunohistochemistry

Formalin-fixed, paraffin-embedded nude mice xenograft tumor sections were deparaffinized in xylene and rehydrated through graded alcohols. Endogenous peroxidase was blocked with 3% H_2O_2. Antigen retrieval was performed via microwave treatment in 0.01 mol/L citrate sodium buffer (pH 6.0). The sections were incubated overnight at 4 °C with primary antibodies, followed by detection using the Envision + Dual Link System-HRP DAB kit (Dako, Carpinteria, CA, USA). The sections were counterstained with hematoxylin. The primary antibodies are summarized in **Supplementary Table 3**. Images were captured and scanned using Pannoramic Desk and Pannoramic Midi (3D HISTECH, Budapest, Hungary). Immunohistochemical integral optical density (IOD) was analyzed using ImageJ software (National Institutes of Health, Bethesda, MD, USA), and the average optical density (IOD/area) was calculated.

Supplementary Table 1. Clinicopathological characteristics of ESCC patients

Clinicopathological characteristics	In situ hybridization Cohort (n=76)<br>N (%)
Age (years) (32-74 years; median 58 years)	
≤60	50 (65.8)
>60	26 (34.2)
Sex	
Male	59 (77.6)
Female	17 (22.4)
Tumor length (cm)	
<5	60 (78.9)
≥5	16 (21.1)
Tumor location	
Upper	7 (9.2)
Middle	40 (52.6)
Lower	29 (38.2)
Tumor differentiation	
Well	12 (15.8)
Moderate	40 (52.6)
Poor	24 (31.6)
Pathologic T stage	
T1	16 (21.1)
T2	15 (19.7)
T3	39 (51.3)
T4	6 (7.9)
Pathologic N stage	
N0	32 (42.1)
N1	25 (32.9)
N2	14 (18.4)
N3	5 (6.6)
Pathologic TNM stage	
I	11 (14.5)
II	24 (31.6)
III	33 (43.4)
IV	8 (10.5)
Adjuvant therapy	
No	35 (46.1)
Yes	
Radiotherapy	9 (11.8)
Chemotherapy	25 (32.9)
Chemoradiotherapy	7 (9.2)

Supplementary Table 2. Data of sequences for cell transfection and qPCR in this study

Gene	Sequence
si-PTEN	Sence: 5’-GAAGGCGUAUACAGGAACATT-3’<br>Anti-sence: 5’-UGUUCCUGUAUACGCCUUCTT-3’
si-control	Sence: 5’-UUCUCCGAACGUGUCACGUTT-3’<br>Anti-sence: 5’-ACGUGACACGUUCGGAGAATT-3’
Cel-miR-39	5’-TCACCGGGTGTAAATCAGCTTG-3’
hsa-miR-193a-3p	5’-AACTGGCCTACAAAGTCCCAGT-3’
hsa-miR-197-3p	5’-TTCACCACCTTCTCCACCCAGC-3’
hsa-miR-376c-3p	5’-AACATAGAGGAAATTCCACGT-3’
hsa-miR-380-3p	5’-TATGTAATATGGTCCACATCTT-3’
PTEN	Forward: 5’-TGGATTCGACTTAGACTTGACCT-3’<br>Reverse: 5’-GGTGGGTTATGGTCTTCAAAAGG-3’
GAPDH	Forward: 5’-ACAACTTTGGTATCGTGGAAGG-3’<br>Reverse: 5’-GCCATCACGCCACAGTTTC-3’

Supplementary Table 3. Antibodies

Antibody	WB	IF	IHC	Company
α-SMA<br>(ab124964)	-	1:500	-	Abcam, Cambridge, MA, USA
FAP<br>(15384-1-AP)	-	1:200	-	Proteintech, Chicago, USA
FSP-1<br>(ab124805)	-	1:100	-	Abcam, Cambridge, MA, USA
CD63<br>(ab134045)	1:1000	-	-	Abcam, Cambridge, MA, USA
CD9<br>(ab263019)	1:1000	-	-	Abcam, Cambridge, MA, USA
TSG101<br>(ab125011)	1:5000	-	-	Abcam, Cambridge, MA, USA
PTEN<br>(ab267787)	1:1000	-	1:2000	Abcam, Cambridge, MA, USA
Akt<br>(4691S)	1:1000	-	-	Cell Signaling Technology, Boston, MA, USA
phosphorylated-Akt<br>(4060S)	1:2000	-	1:200	Cell Signaling Technology, Boston, MA, USA
E-cadherin<br>(3195T)	1:1000	-	1:400	Cell Signaling Technology, Boston, MA, USA
Snail<br>(3879T)	1:1000	-	-	Cell Signaling Technology, Boston, MA, USA
Snail<br>(NBP1-80022)	-	-	1:100	Novus Biologicals, Littleton, CO, USA
Vimentin<br>(5741T)	1:1000	-	-	Cell Signaling Technology, Boston, MA, USA
GAPDH<br>(10494-1-AP)	1:5000	-	-	Proteintech, Chicago, USA

Supplementary Table 4. Associations between miR-193a-3p expression levels and the clinicopathological characteristics in patients with ESCC

Clinicopathological characteristics	N	miR-193a-3p levels		
		Low	High	*P* value
Age				
≤ 60	50	18	32	0.239
> 60	26	13	13	
Sex				
Male	59	22	37	0.247
Female	17	9	8	
Tumor length (cm)				
< 5	60	25	35	0.763
≥ 5	16	6	10	
Tumor location				
Upper	7	5	2	0.075
Middle	40	18	22	
Lower	29	8	21	
Tumor differentiation				
Well + Moderate	52	20	32	0.543
Poor	24	11	13	
Pathologic T stage				
T1 + T2	31	18	13	**0.011**
T3 + T4	45	13	32	
Pathologic N stage				
N0 + N1	57	27	30	**0.043**
N2 + N3	19	4	15	
Pathologic TNM stage				
I + II	35	20	15	**0.007**
III + IV	41	11	30	

Supplementary Table 5. Univariate and multivariate Cox proportional-hazards model for DFS in patients with ESCC (n=76)

Variable	Univariate analysis		Multivariate analysis	
	HR (95% CI)	*P*	HR (95% CI)	*P*
Age, year (≤60 vs >60)	0.629 (0.373-1.059)	0.081	0.360 (0.199-0.654)	<0.001
Sex (male vs female)	1.190 (0.632-2.240)	0.591		
Tumor location				
Lower	2.147 (0.740-6.225)	0.160		
Middle	2.103 (0.744-5.946)	0.161		
Upper	Ref	-		
Tumor differentiation				
Poor	1.875 (0.824-4.272)	0.134		
Moderate	1.849 (0.848-4.033)	0.122		
Well	Ref	-		
Pathologic T stage (T1 + T2 vs T3 + T4)	0.465 (0.273-0.793)	0.005	0.721 (0.396-1.314)	0.285
Tumor length, cm (<5 vs ≥5)	0.856 (0.463-1.583)	0.620		
Pathologic N stage (N0 + N1 vs N2 + N3)	0.313 (0.175-0.558)	<0.001	0.249 (0.122-0.506)	<0.001
Adjuvant therapy (Yes vs No)	1.354 (0.812-2.257)	0.245		
miR-193a-3p expression (low vs high)	0.440 (0.257-0.751)	0.003	0.420 (0.239-0.736)	0.002

Supplementary Table 6. Univariate and multivariate Cox proportional-hazards model for DMFS in patients with ESCC (n=76)

Variable	Univariate analysis		Multivariate analysis	
	HR (95% CI)	*P*	HR (95% CI)	*P*
Age, year (≤60 vs >60)	0.645 (0.381–1.091)	0.102		
Sex (male vs female)	0.927 (0.492–1.748)	0.815		
Tumor location				
Lower	2.763 (0.829–9.215)	0.098	2.530 (0.713–8.970)	0.151
Middle	2.925 (0.896–9.545)	0.075	2.664 (0.795–8.935)	0.112
Upper	Ref	-	Ref	-
Tumor differentiation				
Poor	1.919 (0.837–4.397)	0.124	1.885 (0.819–4.338)	0.136
Moderate	1.941 (0.891–4.230)	0.095	2.774 (1.150–6.693)	0.023
Well	Ref	-	Ref	-
Pathologic T stage (T1 + T2 vs T3 + T4)	0.456 (0.266–0.785)	0.005	0.697 (0.376–1.293)	0.252
Tumor length, cm (<5 vs ≥5)	1.011 (0.545–1.873)	0.973		
Pathologic N stage (N0 + N1 vs N2 + N3)	0.301 (0.168–0.540)	<0.001	0.420 (0.218–0.812)	0.010
Adjuvant therapy (Yes vs No)	1.409 (0.841–2.361)	0.192		
miR-193a-3p expression (low vs high)	0.391 (0.227–0.675)	<0.001	0.431 (0.234–0.795)	0.007

Supplementary Table 7. The treatment regimen for the 32 patients who received concurrent definitive chemoradiation therapy

Treatment characteristics	N (%)
Radiation dose (Gy)	
≤ 61.2	27 (84.4)
> 61.2	5 (15.6)
fractionated dose (Gy)	
≤ 2	30 (93.8)
> 2	2 (6.3)
Chemotherapy regimen	
Paclitaxel + carboplatin	29 (90.6)
Paclitaxel + cisplatin	3 (9.4)
